# Safety and immunogenicity of 2-dose heterologous Ad26.ZEBOV, MVA-BN-Filo Ebola vaccination in healthy and HIV-infected adults: A randomised, placebo-controlled Phase II clinical trial in Africa

**DOI:** 10.1371/journal.pmed.1003813

**Published:** 2021-10-29

**Authors:** Houreratou Barry, Gaudensia Mutua, Hannah Kibuuka, Zacchaeus Anywaine, Sodiomon B. Sirima, Nicolas Meda, Omu Anzala, Serge Eholie, Christine Bétard, Laura Richert, Christine Lacabaratz, M. Juliana McElrath, Stephen De Rosa, Kristen W. Cohen, Georgi Shukarev, Cynthia Robinson, Auguste Gaddah, Dirk Heerwegh, Viki Bockstal, Kerstin Luhn, Maarten Leyssen, Macaya Douoguih, Rodolphe Thiébaut, the EBL2002 Study group

**Affiliations:** 1 Centre MURAZ, Bobo-Dioulasso, Burkina Faso; 2 KAVI—Institute of Clinical Research University of Nairobi, Nairobi, Kenya; 3 Makerere University—Walter Reed Project, Kampala, Uganda; 4 Medical Research Council/Uganda Virus Research Institute and London School of Hygiene and Tropical Medicine Uganda Research Unit, Entebbe, Uganda; 5 Centre National de Recherche et de Formation sur le Paludisme (CNRFP), Unité de Recherche Clinique de Banfora, Ouagadougou, Burkina Faso; 6 Unit of Infectious and Tropical Diseases, BPV3, Treichville University Teaching Hospital, Abidjan, Côte d’Ivoire; 7 Univ. Bordeaux, Inserm, Bordeaux Population Health Research Center, UMR 1219; Inria SISTM team; CHU Bordeaux; CIC 1401, EUCLID/F-CRIN Clinical Trials Platform, F-33000, Bordeaux, France; 8 Vaccine Research Institute (VRI), Créteil, France; 9 Université Paris-Est Créteil, Faculté de Médecine, INSERM U955, Team 16, Créteil, France; 10 Vaccine and Infectious Disease Division, Fred Hutchinson Cancer Research Center, Seattle, Washington, United States of America; 11 Janssen Vaccines and Prevention, Leiden, the Netherlands; 12 Janssen Research & Development, Beerse, Belgium; Burnet Institute, AUSTRALIA

## Abstract

**Background:**

We investigated safety, tolerability, and immunogenicity of the heterologous 2-dose Ebola vaccination regimen in healthy and HIV-infected adults with different intervals between Ebola vaccinations.

**Methods and findings:**

In this randomised, observer-blind, placebo-controlled Phase II trial, 668 healthy 18- to 70-year-olds and 142 HIV-infected 18- to 50-year-olds were enrolled from 1 site in Kenya and 2 sites each in Burkina Faso, Cote d’Ivoire, and Uganda. Participants received intramuscular Ad26.ZEBOV followed by MVA-BN-Filo at 28-, 56-, or 84-day intervals, or saline. Females represented 31.4% of the healthy adult cohort in contrast to 69.7% of the HIV-infected cohort. A subset of healthy adults received booster vaccination with Ad26.ZEBOV or saline at Day 365. Following vaccinations, adverse events (AEs) were collected until 42 days post last vaccination and serious AEs (SAEs) were recorded from signing of the ICF until the end of the study. The primary endpoint was safety, and the secondary endpoint was immunogenicity. Anti-Ebola virus glycoprotein (EBOV GP) binding and neutralising antibodies were measured at baseline and at predefined time points throughout the study.

The first participant was enrolled on 9 November 2015, and the date of last participant’s last visit was 12 February 2019. No vaccine-related SAEs and mainly mild-to-moderate AEs were observed among the participants. The most frequent solicited AEs were injection-site pain (local), and fatigue, headache, and myalgia (systemic), respectively. Twenty-one days post-MVA-BN-Filo vaccination, geometric mean concentrations (GMCs) with 95% confidence intervals (CIs) of EBOV GP binding antibodies in healthy adults in 28-, 56-, and 84-day interval groups were 3,085 EU/mL (2,648 to 3,594), 7,518 EU/mL (6,468 to 8,740), and 7,300 EU/mL (5,116 to 10,417), respectively. In HIV-infected adults in 28- and 56-day interval groups, GMCs were 4,207 EU/mL (3,233 to 5,474) and 5,283 EU/mL (4,094 to 6,817), respectively. Antibody responses were observed until Day 365. Ad26.ZEBOV booster vaccination after 1 year induced an anamnestic response.

Study limitations include that some healthy adult participants either did not receive dose 2 or received dose 2 outside of their protocol-defined interval and that the follow-up period was limited to 365 days for most participants.

**Conclusions:**

Ad26.ZEBOV, MVA-BN-Filo vaccination was well tolerated and immunogenic in healthy and HIV-infected African adults. Increasing the interval between vaccinations from 28 to 56 days improved the magnitude of humoral immune responses. Antibody levels persisted to at least 1 year, and Ad26.ZEBOV booster vaccination demonstrated the presence of vaccination-induced immune memory. These data supported the approval by the European Union for prophylaxis against EBOV disease in adults and children ≥1 year of age.

**Trial registration:**

ClinicalTrials.gov NCT02564523

## Abstract 2

Houreratou Barry and co-workers report on safety and immunogenicity of an Ebola vaccine in adults across four African countries.

## Author summary

**Why was this study done?:**

- With Ebola outbreaks increasing, there is an unmet medical need for a prophylactic vaccine to prevent and mitigate Ebola outbreaks.
- To address the urgent medical need during the 2014 to 2016 outbreak, the clinical development of the 2-dose vaccine regimen comprising of Ad26.ZEBOV and MVA-BN-Filo was accelerated. This Phase II study was part of this accelerated program, evaluating the safety and immunogenicity of the 2-dose vaccine regimen in healthy and HIV-infected African adults, with 28-, 56-, and 84-day intervals between doses.
- The study was amended to evaluate safety and immunogenicity of a booster vaccination with Ad26.ZEBOV, administered approximately 1 year after the first vaccination, in healthy adults.

**What did the researchers do and find?:**

- We conducted a randomised trial to assess the safety and the immunogenicity of the Ad26.ZEBOV, MVA-BN-Filo vaccine regimen in 3 different vaccination intervals in healthy and HIV-infected adults.
- The vaccine regimen was well tolerated and induced marked immune responses; the highest humoral responses were observed after vaccination with 56-day and 84-day intervals.
- Anamnestic responses were observed in all healthy participants receiving Ad26.ZEBOV as booster at 1 year after the first dose.

**What do these findings mean?:**

- Our results demonstrate that the Ad26.ZEBOV, MVA-BN-Filo vaccine regimen is safe and immunogenic in healthy and HIV-infected adults and induces immune memory that can rapidly be reactivated.
- Our findings support the prophylactic use of the 2-dose Ad26.ZEBOV, MVA-BN-Filo vaccine regimen against Ebola infection in African adult populations.
- The 2-dose vaccine regimen comprising of Ad26.ZEBOV and MVA-BN-Filo has received marketing authorisation under exceptional circumstances for prophylactic use against EVD in adults and children ≥1 year old within the European Union.

## Introduction

Since its first identification as the cause of a haemorrhagic fever in 1976, Ebola virus (EBOV) has become increasingly recognised as a widely dispersed pathogen across the African continent [1], with an increasing number of outbreaks. In most cases, infection starts with nonspecific influenza-like symptoms—fatigue, myalgia, arthralgia, headache, and fever. These may be followed by more severe clinical manifestations ultimately leading to multi-organ dysfunction, vascular leakage, and intense generalised bleeding resulting in death within 5 to 8 days of symptom onset [2]. In the biggest outbreak to date in Guinea, Liberia and Sierra Leone in 2014 to 2016, there were 28,616 cases and 11,310 deaths [3]. The 10th outbreak in Democratic Republic of the Congo (DRC) from 2018 to 2020 in the Kivu area was the second largest in history with 3,470 cases, including 2,287 deaths [4,5]. Several EBOV vaccines are in development [6] and target the viral surface glycoprotein (GP). The main difference between vaccines is the vector used to deliver the antigen. In a ring vaccination strategy, efficacy is assessed through the vaccination of contacts, and contacts of contacts, of individuals with confirmed cases of EBOV disease. This strategy was applied in the 2014 to 2016 West African outbreak in Guinea and the 2018 to 2020 DRC outbreak, in which 1 dose of a recombinant replication-competent vesicular stomatitis viral vectored vaccine (rVSV-ZEBOV-GP) expressing the GP of the Kikwit variant of the Zaire species demonstrated 100% [7] and 97.5% [8] efficacy from 10 days after vaccination, respectively. This vaccine (Ervebo) has been approved by the Food and Drug Administration (FDA) [9] for use in adults ≥18 years old and received conditional approval by the European Medicines Agency [10]. It was prequalified by the World Health Organization (WHO) [11] and is recommended by the WHO Strategic Advisory Group of Experts on Immunization (SAGE) for high-risk populations in response to the 2018 to 2020 outbreak in the DRC [12].

Recently, the heterologous 2-dose regimen of Ad26.ZEBOV (Zabdeno) and MVA-BN-Filo (Mvabea) received approval by the European Commission under exceptional circumstances for use in children and adults [13]. This regimen is recommended by WHO SAGE for prophylaxis and evaluation in lower-risk populations [12]. Following WHO SAGE recommendation, the heterologous regimen is being studied in the DRC and in an ongoing vaccination campaign in Rwanda in response to the 2018 to 2020 outbreak [14].

In Phase I studies, this regimen had an acceptable safety profile and was immunogenic in healthy 18- to 50-year-old volunteers in England [15], Kenya [16], and Tanzania and Uganda [17], inducing both humoral and cellular immune responses against EBOV GP. These data encouraged further evaluation in Phase II and III studies.

We present a Phase II study to determine the safety, tolerability, and immunogenicity of this heterologous 2-dose regimen, with various intervals between the 2 doses in the target population, i.e., healthy African adults, adolescents, and children. We also included a cohort of HIV-infected adults to ensure that the vaccine regimen is safe and immunogenic in this population. Finally, we explored the effect of an Ad26.ZEBOV booster vaccination administered 1 year post-dose 1 in a subset of healthy adults. This paper presents the results from the healthy and HIV-infected adult cohorts. Data from the adolescent and paediatric populations will be published separately.

## Methods

### Study overview

This Phase II randomised, observer-blind, placebo-controlled study was conducted in 7 sites in Africa [Burkina Faso (Bobo-Dioulasso, Banfora); Côte d’Ivoire (Abidjan, Toupah/Ousrou); Kenya (Nairobi); Uganda (Masaka, Kampala)] between November 2015 and February 2019. The protocol was approved by local and national independent Ethics Committees and Institutional Review Boards, and the study was done according to the Declaration of Helsinki and International Conference on Harmonisation Good Clinical Practice Guidelines. All adult participants supplied written informed consent before enrolment. An independent data monitoring committee was established to assess the safety data regularly during the study. The study was registered at ClinicalTrials.gov NCT02564523. The study protocol, including the CONSORT checklist, can be found in **S1 Study Protocol** and **S1 CONSORT Checklist**.

The primary objective was to assess the safety and tolerability of 3 vaccination schedules with Ad26.ZEBOV administered on Day 1 and MVA-BN-Filo on Days 29, 57, or 85 in healthy African adults, and of 2 schedules with Ad26.ZEBOV on Day 1 and MVA-BN-Filo on Days 29 or 57 in HIV-infected African adults. Secondary objectives included immunogenicity assessments such as EBOV GP–specific immunoglobulin G (IgG) binding antibodies 21 days post-dose 2 in healthy and HIV-infected adults, and the safety and tolerability of an Ad26.ZEBOV booster dose on Day 365 in a subset of 90 healthy adults. Exploratory objectives included assessments of binding antibodies at other time points in healthy (including after the booster dose) and HIV-infected adults and measurement of EBOV GP–specific neutralising antibodies and cellular immune responses in subsets of healthy adults.

### Study participants, randomisation, and blinding

Study participants were recruited from the general population and from HIV–positive cohorts in Africa. Information regarding the study was shared through community meetings, posters, and public conferences where volunteers were invited to study sites. After screening by the investigators at study sites, eligible healthy adults aged 18 to 70 years were randomised to 3 groups (1:1:1) in parallel using randomly permuted blocks. Groups 1, 2, and 3 correspond to 28-, 56-, and 84-day intervals, respectively. For the healthy adult cohort, there was stratification by age (18 to 50 years and >50 years). The interactive web response system (IWRS; prepared by Signant Health) assigned a unique code, which dictated the assignment and matching vaccination schedule for a participant. These codes were maintained within the IWRS and were not provided to the investigators. During the study pause (detailed in the results section), investigators were instructed not to proceed with any randomisation until the pause was lifted.

Healthy adults were enrolled in parallel to Groups 1 to 3 until a target of 132 participants had been randomised to Group 3. Further enrolment continued to Groups 1 and 2 with 1:1 randomisation until each group contained 264 participants. When 7-day safety data were available from 25% of healthy adults, enrolment of the HIV-infected cohort started, consisting of 142 HIV-infected adults aged 18 to 50 years randomised 1:1 to 2 groups to receive the 2 vaccinations with a 28- or 56-day interval. Participants in each of the 5 groups in healthy and HIV-infected cohorts were further randomised 5:1 to receive active vaccines or placebo in the different regimens. The first 90 healthy adult participants in Groups 1 and 2 (vaccinees, *n =* 73 or placebo, *n* = 17) who provided consent received an Ad26.ZEBOV booster dose or placebo on Day 365. Participants, investigators, and study staff remained blinded to the allocation of investigational products throughout the study. Vaccines and placebo were prepared by a site pharmacist who was the only unblinded member of staff. The pharmacist received the randomisation number and allocated the right study vaccine to the participant. Masking tape was used to cover the dispensing syringes containing the study vaccine/placebo allocated to each study participant.

Eligible volunteers were confirmed healthy based on physical examination, ECG, vital signs, and clinical laboratory assessments performed at screening and their medical history. HIV-infected volunteers had to have been diagnosed at least 6 months previously, have a CD4+ cell count >350 cells/μL, be in an otherwise reasonably good medical condition without an AIDS-defining diagnosis or a clinically significant disease, and be on a stable regimen of antiretroviral therapy. Full details of inclusion and exclusion criteria are supplied in **S1 Text**.

### Sample size determination

The overall planned sample size for healthy and HIV-infected cohorts included 792 participants who were to receive either the 2-dose heterologous vaccination regimen of Ad26.ZEBOV and MVA-BN-Filo or placebo to substantially contribute to the overall safety database of the regimen. In the healthy adult cohort, a total of 550 participants were to receive Ad26.ZEBOV and MVA-BN-Filo across the different vaccination schedules (220 participants in 28-day interval and 220 participants in 56-day interval; 110 in 84-day interval); 110 participants were to receive placebo. The preplanned smaller sample size target for Group 3 (84-day interval) was selected to provide preliminary information on the effect of a longer interval between doses. The larger sample size target for Groups 1 and 2 was reflective of the schedules for which an indication was being sought. As the study was not powered to test statistical difference between the groups, we consider that these differences in recruitment are unlikely to impact the conclusions of the study. In the HIV-infected adult cohort, a total of 110 participants were to receive Ad26.ZEBOV and MVA-BN-Filo across the different vaccination schedules (55 participants in 28-day interval and 55 in 56-day interval); 22 participants were to receive placebo. Sample size determination was not based on formal statistical hypothesis testing. However, in case a specific adverse event (AE) was not observed, the one-sided 97.5% upper confidence limit of the true incidence rate of this AE was less than 6.5%, 3.3%, 1.7%, and 0.7% for a sample size of 55, 110, 220, and 550 participants, respectively.

### Vaccines

Ad26.ZEBOV is a monovalent, recombinant, replication-incompetent adenovirus 26-vectored vaccine encoding EBOV Mayinga variant GP. MVA-BN-Filo is a multivalent, recombinant, nonreplicating, modified vaccinia Ankara-vectored vaccine encoding EBOV Mayinga, Sudan virus Gulu, and Marburg virus Musoke variant GPs, as well as Taï Forest virus nucleoprotein [15–17].

Ad26.ZEBOV vaccination with 5 × 10^10^ viral particles on Day 1 was followed by 1 × 10^8^ infectious units of MVA-BN-Filo on Day 29 (Group 1), Day 57 (Group 2), or Day 85 (Group 3). The booster vaccination (Day 365) consisted of Ad26.ZEBOV 5 × 10^10^ viral particles or placebo. Both vaccines and placebo (0.9% saline) were administered by intramuscular injection (0.5 mL) in the deltoid. The second injection was administered in the opposite arm to the first injection.

### Safety and tolerability assessments

All participants were assessed at 30 and 60 minutes after each vaccination for any immediate AEs. Participants were supplied with diary cards, rulers, and thermometers to measure and record solicited local and systemic AEs and daily body temperature each evening for 7 days. AEs were graded as 1 (mild), 2 (moderate), or 3 (severe) according to the adapted Division of AIDS (DAIDS) Table (2017) [18]. Laboratory assessments of haematology and serum chemistry were done at screening, prevaccination, and 7 days after vaccination and graded according to the FDA toxicity grading scale for healthy adult and adolescent volunteers enrolled in preventive vaccine clinical trials [19]. Any unsolicited AEs, until 42 days after the second vaccination or 28 days after the booster dose, were documented by the investigator. Any serious AEs (SAEs) were recorded throughout the study, with follow-up to 1 year after the last vaccination or event resolution as appropriate.

### Immunogenicity assessments

Serum samples obtained from all participants at 4 time points were used to assess humoral immune responses as binding IgG antibodies specific to EBOV GP: before the first vaccination on Day 1 and the second vaccination on Days 29, 57 or 85, and 21 days post-dose 2, with a final sample on Day 365 to assess persistence of antibodies. In the booster subset, additional blood samples were obtained 4, 7, 21, and 365 days after receiving the booster dose. EBOV GP–specific binding antibodies were measured at all time points using an EBOV GP Filovirus Animal Non-Clinical Group (FANG) enzyme-linked immunosorbent assay (ELISA) [20] performed at Q^2^ Solutions (San Juan Capistrano, CA, USA).

Virus neutralising antibodies were measured in a subset (19%; *n =* 100/536 [*n* = 50/268 assigned to the 28-day interval regimen; *n* = 50/268 assigned to the 56-day interval regimen]) of healthy adults on Day 1, 21 days after MVA-BN-Filo and at 1 year using the EBOV GP pseudovirion neutralisation assay (psVNA) at Monogram (San Francisco, CA, USA) (**S2 and S3 Texts**). Levels of neutralising antibodies to the Ad26 vector were measured using an Ad26-specific virus neutralisation assay (Ad26 VNA) in a subset (19%; *n =* 100/536 [*n* = 50/268 assigned to the 28-day interval regimen; *n* = 50/268 assigned to the 56-day interval regimen]) of healthy adults and in all HIV-infected participants before vaccination on Day 1 and again on Day 365 (pre-booster) for healthy adults (Janssen Vaccines and Prevention, Leiden, the Netherlands). Intracellular cytokine staining (ICS) and interferon gamma (IFNγ) enzyme-linked immunospot (ELISpot) assays were done in a subset of healthy (16%; *n =* 84/536 [*n* = 46/268 assigned to the 28-day interval regimen; *n* = 38/268 assigned to the 56-day interval regimen]) and HIV-infected (21%; *n* = 15/71 assigned to the 56-day interval regimen) participants (see **S2 Text** for selection criteria) on Day 1, 21 days post-dose 2 and 1 year post-dose 1 (HIV Vaccine Trials Network, Seattle, WA, USA), as previously described [15–17,21].

### Statistical analyses

The study was originally designed as a prospective study with no formal hypothesis testing. Safety analyses were descriptive, based on the full analysis set consisting of all participants who received at least 1 dose of active vaccine or placebo regardless of protocol deviations. The main immunogenicity analysis is based on the per-protocol analysis set. It included all randomised and vaccinated participants who received both vaccinations in the protocol-defined windows, had at least 1 evaluable immunogenicity sample post vaccination, and no major protocol deviations influencing the immune response. A sensitivity analysis was conducted for those who received their second vaccination outside the protocol-defined windows. These participants were part of the immunogenicity analysis set that included all randomised and vaccinated participants who had at least 1 postvaccination (i.e., after date of vaccination) evaluable immunogenicity sample. Binding antibody results were expressed as group geometric mean concentrations (GMCs) of ELISA units with 95% confidence intervals (CIs) at each time point and responder rates, i.e., the percentage of each group with post-vaccination concentrations >2.5-fold the lower limit of quantification [LLOQ; 36.11 ELISA units/mL (EU/mL)] in baseline seronegative individuals or >2.5-fold the baseline value in initially seropositive participants. All values below the LLOQ were imputed with half the LLOQ value. Exploratory analysis responder definitions are reported in **S4 Text**. A Spearman correlation coefficient was calculated for EBOV GP–specific binding antibody concentrations (FANG ELISA) and psVNA titres 21 days post-dose 2.

While no formal statistical testing was originally planned, statistical analyses were performed post hoc for primary outcomes. The statistical significance was set at *p*-value < 0.05. No test multiplicity adjustments were generally performed.

## Results

### Participant enrolment and clinical hold

Enrolment of participants began on 9 November 2015 and was paused on 28 April 2016 to permit a thorough investigation of 2 SAEs that occurred in a concomitant study in Europe [22]. At the time of the pause, more than 200 healthy participants had been enrolled, some awaiting their first vaccination, and some already having received the first vaccine. When the independent data monitoring committee and the regulatory authorities agreed to continuation of the study (clinical hold lifted by US FDA on 16 June 2016), participants who had already received 1 dose before the clinical hold were offered the second vaccination out-of-window but were excluded from the per-protocol analysis set. Those who had been enrolled but had not received their first vaccination were rescreened and vaccinated according to their preassigned study schedule. Vaccination of the HIV-infected participants only started after lifting the clinical hold. Monitoring was continued for all participants up to the 365-day follow-up, irrespective of their inclusion in the per-protocol analysis set. The date of last participant last visit was 12 February 2019.

### Baseline demographics

**Fig 1:**
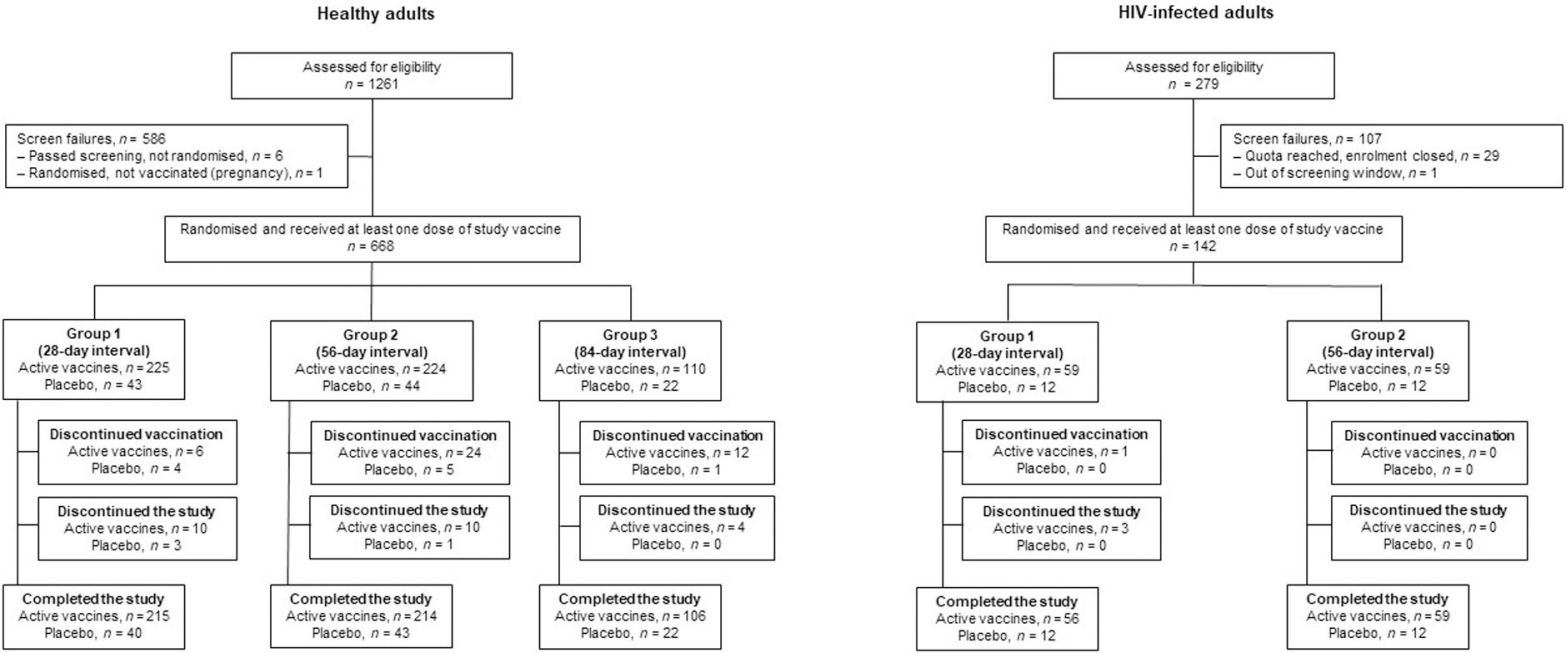
Study disposition of healthy adults and HIV-infected adults. *n =* number of participants.

The full analysis set included 668 healthy adults (559 vaccinees, 109 placebo) and 142 HIV-infected adults (118 vaccinees, 24 placebo), who received at least 1 dose of Ad26.ZEBOV or placebo (**Fig 1**). Participant demographic data are shown in **Table 1**. Among these, 400 healthy adults and 140 HIV-infected adults were included in the per-protocol analysis set (**Table 1**).

**Table 1:** Baseline characteristics of the participants in the full analysis set.*

	Healthy adults						HIV-infected adults			
	Group 1: 28-day interval		Group 2: 56-day interval		Group 3: 84-day interval		Group 1: 28-day interval		Group 2: 56-day interval	
Characteristic	Active vaccines	Placebo	Active vaccines	Placebo	Active vaccines	Placebo	Active vaccines	Placebo	Active vaccines	Placebo
N =	225	43	224	44	110	22	59	12	59	12
Sex, n (%)										
Male	152 (67.6)	28 (65.1)	153 (68.3)	28 (63.6)	83 (75.5)	14 (63.6)	20 (33.9)	4 (33.3)	17 (28.8)	2 (16.7)
Female	73 (32.4)	15 (34.9)	71 (31.7)	16 (36.4)	27 (24.5)	8 (36.4)	39 (66.1)	8 (66.7)	42 (71.2)	10 (83.3)
Ethnicity^‡^, n (%)										
Black	223 (99.1)	42 (97.7)	224 (100)	43 (97.7)	109 (99.1)	22 (100)	59 (100)	12 (100)	59 (100)	12 (100)
Asian	2 (0.9)	1 (2.3)	0	0	1 (0.9)	0	0	0	0	0
White	0	0	0	1 (2.3)	0	0	0	0	0	0
Age (years)										
Mean (SD)	33.3 (12.4)	32.0 (10.4)	33.3 (11.5)	33.3 (11.6)	31.4 (11.5)	33.7 (12.3)	38.8 (6.6)	34.3 (7.7)	39.0 (6.7)	42.1 (5.0)
Median (range)	29.0 (18–69)	30.0 (19–60)	30.0 (18–66)	30.0 (18–65)	27.0 (18–67)	31.0 (19–66)	40.0 (21–49)	36.5 (22–48)	39.0 (18–49)	41.5 (34–50)
Body mass index (kg/m^2^)										
Mean (SD)	23.0 (3.95)	23.4 (4.55)	23.4 (3.65)	22.9 (4.18)	23.0 (4.29)	25.1 (6.10)	24.5 (4.51)	23.0 (3.88)	24.0 (4.51)	24.5 (3.78)
Per-protocol analysis set^#^										
N^#^ =	173	32	137	24	27	7	58	11	59	12

*Participants who received at least 1 dose of the study vaccines or placebo.

^‡^Ethnicity was recorded as race within the database.

^#^The numbers of participants who received the study vaccines or placebo within the predefined time windows and supplied at least 1 postvaccination sample with no protocol deviations.

N, number of participants who received at least 1 dose of the study vaccines or placebo; SD, standard deviation.

### Safety

Safety analyses are based on the full analysis set. There were 27 SAEs (which occurred in 24 participants) including 1 death overall during the study (**Table A in S1 Data**). One HIV-infected participant died due to alcohol poisoning more than 4 weeks after receipt of MVA-BN-Filo. No SAE was considered to be vaccine-related by the investigators.

Both vaccines were well tolerated in healthy and HIV-infected adults (**Table 2**). Reactogenicity results were similar when healthy adults were stratified to 18 to 50 years and >50 years age groups (**Table B in S1 Data**).

**Table 2:** Solicited and unsolicited AEs in the full analysis set after each vaccination dose, no. (%).*

		Healthy adults								HIV-infected adults					
		Group 1: 28-day interval		Group 2: 56-day interval		Group 3: 84-day interval		All groups		Group 1: 28-day interval		Group 2: 56-day interval		All groups	
		**Ad26**	**Placebo**	**Ad26**	**Placebo**	**Ad26**	**Placebo**	**Ad26**	**Placebo**	**Ad26**	**Placebo**	**Ad26**	**Placebo**	**Ad26**	**Placebo**
**Post-dose 1**	N	225	43	224	44	110	22	559	109	59	12	59	12	118	24
Solicited AE, n (%)		161 (71.6)	30 (69.8)	161 (71.9)	31 (70.5)	83 (75.5)	15 (68.2)	405 (72.4)	76 (69.7)	48 (81.4)	7 (58.3)	45 (76.3)	3 (25.0)	93 (78.8)	10 (41.7)
Grade 3^‡^		4 (1.8)	0	6 (2.7)	1 (2.3)	5 (4.5)	1 (4.5)	15 (2.7)	2 (1.8)	1 (1.7)	0	2 (3.4)	0	3 (2.5)	0
Solicited local AE, n (%)		123 (54.7)	16 (37.2)	121 (54.0)	20 (45.5)	63 (57.3)	11 (50.0)	307 (54.9)	47 (43.1)	34 (57.6)	4 (33.3)	35 (59.3)	2 (16.7)	69 (58.5)	6 (25.0)
Grade 3^‡^		0	0	1 (0.4)	0	1 (0.9)	0	2 (0.4)	0	0	0	0	0	0	0
Solicited systemic AE, n (%)		146 (64.9)	27 (62.8)	140 (62.5)	26 (59.1)	75 (68.2)	14 (63.6)	361 (64.6)	67 (61.5)	44 (74.6)	7 (58.3)	36 (61.0)	2 (16.7)	80 (67.8)	9 (37.5)
Grade 3^‡^		4 (1.8)	0	6 (2.7)	1 (2.3)	5 (4.5)	1 (4.5)	15 (2.7)	2 (1.8)	1 (1.7)	0	2 (3.4)	0	3 (2.5)	0
Any unsolicited AE, n (%)		96 (42.7)	17 (39.5)	68 (30.4)	17 (38.6)	37 (33.6)	6 (27.3)	201 (36.0)	40 (36.7)	25 (42.4)	7 (58.3)	25 (42.4)	3 (25.0)	50 (42.4)	10 (41.7)
Grade 3^‡^		16 (7.1)	5 (11.6)	7 (3.1)	4 (9.1)	3 (2.7)	2 (9.1)	26 (4.7)	11 (10.1)	5 (8.5)	2 (16.7)	4 (6.8)	1 (8.3)	9 (7.6)	3 (12.5)
		**MVA**	**Placebo**	**MVA**	**Placebo**	**MVA**	**Placebo**	**MVA**	**Placebo**	**MVA**	**Placebo**	**MVA**	**Placebo**	**MVA**	**Placebo**
**Post-dose 2**	N	219	39	200	39	98	21	517	99	58	12	59	12	117	24
Solicited AE, n (%)		152 (69.4)	15 (38.5)	148 (74.0)	25 (64.1)	73 (74.5)	15 (71.4)	373 (72.1)	55 (55.6)	34 (58.6)	5 (41.7)	31 (52.5)	6 (50.0)	65 (55.6)	11 (45.8)
Grade 3^‡^		5 (2.3)	2 (5.1)	2 (1.0)	1 (2.6)	6 (6.1)	0	13 (2.5)	3 (3.0)	0	0	0	1 (8.3)	0	1 (4.2)
Solicited local AE, n (%)		126 (57.5)	9 (23.1)	113 (56.5)	16 (41.0)	57 (58.2)	9 (42.9)	296 (57.2)	34 (34.3)	26 (44.8)	2 (16.7)	25 (42.4)	2 (16.7)	51 (43.6)	4 (16.7)
Grade 3^‡^		2 (0.9)	0	0	0	2 (2.0)	0	4 (0.8)	0	0	0	0	0	0	0
Solicited systemic AE, n (%)		121 (55.3)	14 (35.9)	121 (60.5)	21 (53.8)	64 (65.3)	14 (66.7)	306 (59.2)	49 (49.5)	32 (55.2)	4 (33.3)	26 (44.1)	6 (50.0)	58 (49.6)	10 (41.7)
Grade 3^‡^		4 (1.8)	2 (5.1)	2 (1.0)	1 (2.6)	5 (5.1)	0	11 (2.1)	3 (3.0)	0	0	0	1 (8.3)	0	1 (4.2)
Any unsolicited AE, n (%)		74 (33.8)	15 (38.5)	58 (29.0)	13 (33.3)	34 (34.7)	7 (33.3)	166 (32.1)	35 (35.4)	22 (37.9)	4 (33.3)	22 (37.3)	4 (33.3)	44 (37.6)	8 (33.3)
Grade 3^‡^		5 (2.3)	1 (2.6)	7 (3.5)	2 (5.1)	3 (3.1)	1 (4.8)	15 (2.9)	4 (4.0)	1 (1.7)	0	3 (5.1)	1 (8.3)	4 (3.4)	1 (4.2)
		**Ad26**	**Placebo**	**Ad26**	**Placebo**			**Ad26**	**Placebo**						
**Post-booster**	N	34	8	39	9	NA	NA	73	17						
Solicited AE, n (%)		20 (58.8)	3 (37.5)	22 (56.4)	4 (44.4)			42 (57.5)	7 (41.2)						
Grade 3^‡^		0	0	1 (2.6)	0			1 (1.4)	0						
Solicited local AE, n (%)		18 (52.9)	1 (12.5)	16 (41.0)	3 (33.3)			34 (46.6)	4 (23.5)						
Grade 3^‡^		0	0	0	0			0	0						
Solicited systemic AE, n (%)		17 (50.0)	2 (25.0)	18 (46.2)	4 (44.4)			35 (47.9)	6 (35.3)						
Grade 3^‡^		0	0	1 (2.6)	0			1 (1.4)	0						
Any unsolicited AE, n (%)		9 (26.5)	3 (37.5)	14 (35.9)	0			23 (31.5)	3 (17.6)						
Grade 3^‡^		0	0	3 (7.7)	0			3 (4.1)	0						

*Solicited local and systemic AEs were recorded on diary cards by participants for 7 days and unsolicited AEs until Day 28 after each vaccination. Solicited local AEs included pain, erythema, swelling, and pruritis; systemic AEs included nausea/vomiting, fatigue/malaise, headache, myalgia, arthralgia, and chills.

^‡^Grade 3 was defined as symptoms causing inability to perform usual social and functional activities.

Vaccines: Ad26 = Ad26.ZEBOV at a dose of 5 × 10^10^ vp; MVA = MVA-BN-Filo at a dose of 1 × 10^8^ Inf.U.

AE, adverse event; N, number of participants with data at that time point; n (%), number (percentage) of participants with one or more events; NA, not applicable.

#### Safety of 2-dose vaccination primary regimen

Overall, in healthy adults, solicited local AEs were reported in 307/559 (54.9%), 296/517 (57.2%), 47/109 (43.1%), and 34/99 (34.3%) participants following Ad26.ZEBOV, MVA-BN-Filo, first placebo, and second placebo injections, respectively. The most common solicited local AE was mild or moderate injection-site pain. In HIV-infected adults, solicited local AEs were reported in 69/118 (58.5%), 51/117 (43.6%), 6/24 (25.0%), and 4/24 (16.7%) participants following Ad26.ZEBOV, MVA-BN-Filo, first placebo, and second placebo injections, respectively. The event rate following Ad26.ZEBOV in healthy adults (54.9%) was not statistically different (*p*-value = 0.54) from that of the HIV-infected adults (58.5%; **Table C in S1 Data**); following MVA-BN-Filo, a significant difference (57.2% versus 43.6%, respectively, *p*-value = 0.01) was observed. In the healthy adults, 2/559 (0.4%) and 4/517 (0.8%) participants reported Grade 3 local AEs following Ad26.ZEBOV and MVA-BN-Filo injections, respectively; no Grade 3 local AEs were reported by placebo recipients or HIV-infected adults.

In healthy adults, solicited systemic AEs were reported in 361/559 (64.6%), 306/517 (59.2%), 67/109 (61.5%), and 49/99 (49.5%) participants following Ad26.ZEBOV, MVA-BN-Filo, first placebo, and second placebo injections, respectively. The most frequent systemic AEs were fatigue, headache, and myalgia. Compared to the healthy adults, there was no significant difference (*p*-value = 0.53 following Ad26.ZEBOV; *p*-value = 0.06 following MVA-BN-Filo; **Table C in S1 Data**) in reactogenicity profile in HIV-infected adults in whom systemic AEs were reported in 80/118 (67.8%), 58/117 (49.6%), 9/24 (37.5%), and 10/24 (41.7%) participants following Ad26.ZEBOV, MVA-BN-Filo, first placebo, and second placebo injections, respectively. Grade 3 systemic AEs were reported in 0% to 4.2% of healthy and HIV-infected adults following Ad26.ZEBOV, MVA-BN-Filo, or placebo injections.

In healthy adults, fever (≥38.0°C) occurred in 25/559 (4.5%), 33/517 (6.4%), 4/109 (3.6%), and 3/99 (3.0%) participants following Ad26.ZEBOV, MVA-BN-Filo, first placebo, and second placebo injections, respectively. Grade 3 fever (>38.9°C) was reported in 4/559 (0.7%), 9/517 (1.7%), 0/109 (0.0%), and 1/99 (1.0%) participants following Ad26.ZEBOV, MVA-BN-Filo, first placebo, and second placebo injections, respectively (**Table D in S1 Data**). In HIV-infected participants, fever was reported in 13/118 (11.0%), 3/117 (2.6%), 2/24 (8.3%), and 3/24 (12.5%) participants following Ad26.ZEBOV, MVA-BN-Filo, first placebo, and second placebo injections, respectively. The fever rate following Ad26.ZEBOV in healthy adults (4.5%) was statistically lower (*p*-value = 0.01) than that of the HIV-infected adults (11.0%; **Table C in S1 Data**); following MVA-BN-Filo, no statistically significant difference (6.4% versus 2.6%, respectively, *p*-value = 0.12) was observed.

Grade 3 fever was reported in 3/118 (2.5%) participants following Ad26.ZEBOV injections, 1/24 (4.2%) participants following second placebo injections and in no participants following MVA-BN-Filo or first placebo injections.

In healthy adults, unsolicited AEs were reported at similar rates in each group; reported in 201/559 (36.0%), 166/517 (32.1%), 40/109 (36.7%), and 35/99 (35.4%) participants following Ad26.ZEBOV, MVA-BN-Filo, first placebo, and second placebo injections, respectively. The most frequent unsolicited AEs were malaria and upper respiratory tract infections (URTIs). The rates of unsolicited AEs reported in HIV-infected adults were 50/118 (42.4%), 44/117 (37.6%), 10/24 (41.7%), and 8/24 (33.3%) in participants following Ad26.ZEBOV, MVA-BN-Filo, first placebo, and second placebo injections, respectively. Compared to the healthy adults, the observed rates in HIV-infected adults were not significantly different following either vaccine (*p*-value = 0.21 following Ad26.EBOV; *p*-value = 0.28 following MVA-BN-filo; **Table C in S1 Data**). The most frequent unsolicited AEs in the HIV-infected adults were URTI and cases of neutropaenia, which were reported equally in vaccine and placebo arms.

#### Safety of booster dose

There was no apparent exacerbation of reactogenicity after the booster dose of Ad26.ZEBOV in healthy adults. Of these 73 participants, 34 (46.6%) and 35 (47.9%) reported local or systemic AEs, respectively, of which only one was Grade 3 (fever).

### Immunogenicity

Overall, immunogenicity results were similar when healthy adults were stratified to 18 to 50 years and >50 years age groups (**Table E in S1 Data**).

#### Binding antibody responses against EBOV GP

The immunogenicity analyses were based on the participants in the per-protocol analysis set. Binding antibodies in all placebo groups were very low or undetectable at all time points.

**Fig 2:**
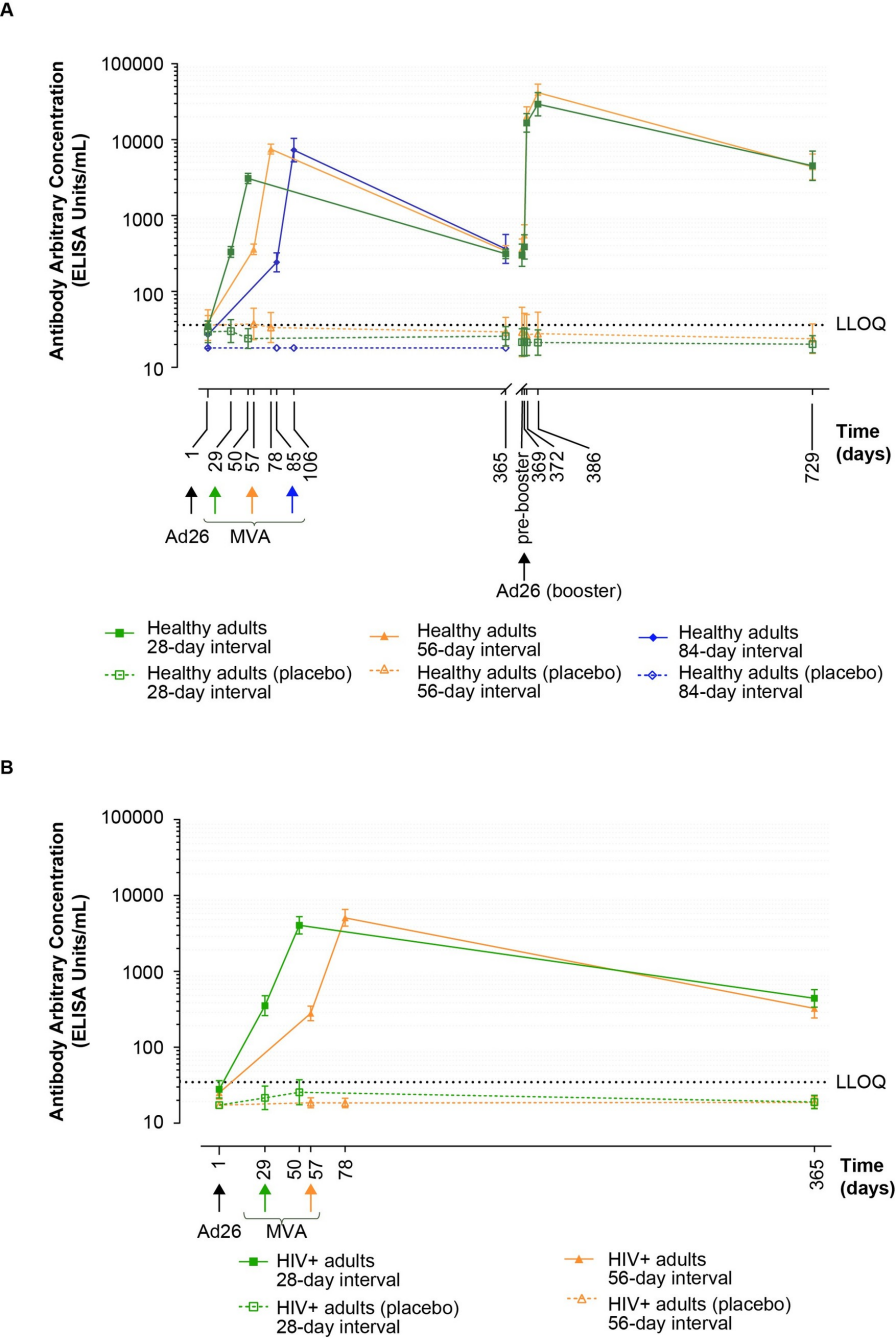
Anti-EBOV GP binding antibody responses. (**A**) Healthy adults administered Ad26.ZEBOV on Day 1 and MVA-BN-Filo 28, 56, or 84 days later as indicated. Subsets of 28- and 56-day groups received Ad26.ZEBOV booster on Day 365. “Pre-booster” indicates the pre-booster vaccination measurement observed in participants who received the booster vaccination. (**B**) HIV-infected adults administered with Ad26.ZEBOV on Day 1 and MVA-BN-Filo 28 or 56 days later, as indicated. Responses are expressed as GMCs (ELISA units/mL, 95% CI). Responses in placebo groups are shown as open symbols. Black dotted line represents the LLOQ. The points (symbols) denote GMCs, and error bars denote 95% CIs. Vaccines: Ad26 = Ad26.ZEBOV at a dose of 5 × 10^10^ vp; MVA = MVA-BN-Filo at a dose of 1 × 10^8^ Inf.U. CI, confidence interval; GMC, geometric mean concentration; LLOQ, lower limit of quantification.

After Ad26.ZEBOV vaccination of healthy adults, there were marked increases in EBOV GP–specific binding antibodies (**Fig 2A**). Prior to dose 2 vaccination, levels at Days 29, 57, and 85 in Groups 1, 2, and 3, respectively, were similar with GMCs of 332 EU/mL (95% CI 282 to 390), 361 EU/mL (95% CI 307 to 423), and 242 EU/mL (95% CI 181 to 323) (**Fig 2A**). The proportion of responders was 77%, 80%, and 81%, respectively (**Table F in S1 Data**). MVA-BN-Filo vaccination elicited further increases in antibody concentrations, reaching GMCs of 3,085 EU/mL (95% CI 2,648 to 3,594), 7,518 EU/mL (95% CI 6,468 to 8,740), and 7,300 EU/mL (95% CI 5,116 to 10,417) in the 28-, 56-, and 84-day interval groups, respectively, 21 days post-dose 2 (**Fig 2A**). The GMCs of binding antibodies were statistically different for the 28- and 56-day interval groups (GMC ratio [95% CI] = 0.4 [0.3 to 0.5], *p*-value < 0.001) and the 28- and 84-day interval groups (GMC ratio [95% CI] = 0.4 [0.3 to 0.7], *p*-value < 0.001) but not for 56- and 84-day interval groups (GMC ratio [95% CI] = 1.0 [0.6 to 1.6], *p*-value = 1.00; **Table G in S1 Data**). At this time, responder rates were 98%, 99%, and 100%, respectively (**Table F in S1 Data**). At Day 365, binding antibodies persisted with GMCs ranging from 313 to 363 EU/mL in 80%, 78%, and 88% of participants in the 28-, 56-, and 84-day groups, respectively (**Fig 2A and Table F in S1 Data**); the GMCs were not statistically different for the interval groups (**Table G in S1 Data**).

In the 28- and 56-day groups of HIV-infected adults, patterns of responses were similar to those in healthy adults in terms of kinetics and magnitude (**Fig 2B**). Prior to dose 2, binding antibody responses were observed in 81% and 88% participants, with GMCs of 368 EU/mL (95% CI 272 to 497) and 291 EU/mL (95% CI 233 to 364) in the 28- and 56-day regimens, respectively (**Table H in S1 Data**). At 21 days post-dose 2, there was no evidence of a statistical difference in GMCs of binding antibodies (GMC ratio [95% CI] = 0.8 [0.6 to 1.1], *p*-value = 0.22) between the groups (**Table G in S1 Data**). The GMCs were 4,207 EU/mL (95% CI 3,233 to 5,474) and 5,283 EU/mL (95% CI 4,094 to 6,817) with 100% responder rates in the 28- and 56-day groups. At Day 365, antibodies persisted in 86% and 88% HIV-infected participants in the 28- and 56-day groups, respectively, with GMCs of 459 EU/mL (95% CI 352 to 600) and 338 EU/mL (95% CI 253 to 450) (**Table H in S1 Data**); no statistically significant difference (GMC ratio [95% CI] = 1.4 [0.9 to 2.0], *p*-value = 0.12) was observed between regimens. Compared to the healthy adults, the GMCs of binding antibodies at 21 days post-dose 2 were statistically different for the HIV-infected adults for both intervals (GMC ratio [95% CI] = 0.7 [0.5 to 0.99], *p*-value = 0.04 for 28-day interval; GMC ratio [95% CI] = 1.4 [1.1 to 1.9], *p*-value = 0.02 for 56-day interval; **Table I in S1 Data**).

#### Response to booster dose

In subsets of healthy participants from the 28- and 56-day interval groups, an Ad26.ZEBOV booster dose on Day 365 elicited anamnestic responses in both groups. GMCs rapidly increased, and, 7 days post-booster, levels were higher than after the initial Ad26.ZEBOV, MVA-BN-Filo regimen; 21 days post-booster, GMCs were 29,315 EU/mL (95% CI 20,614 to 41,689) in the 28-day group and 41,643 EU/mL (95% CI 32,045 to 54,116) in the 56-day group (**Table F in S1 Data**). In both groups, responses persisted in 97% to 100% of participants 1 year post-booster (Day 729) with similar GMCs (95% CI 4,383 to 4,534 EU/mL).

#### Out-of-protocol window

Among healthy participants, 204 (*n =* 169, active; *n* = 35, placebo) received their second dose outside the protocol-defined window. A sensitivity analysis of their binding antibody responses demonstrated that increases in the time interval between first and second doses from 99 to 483 days did not appear to negatively impact the immune response post-dose 2. After Ad26.ZEBOV vaccination, before MVA-BN-Filo vaccination, GMCs were maintained at similar levels for groups in which the interval was extended up to 140 days (266 EU/mL [95% CI 198 to 358]), 196 days (197 EU/mL [95% CI 159 to 245]), 252 days (239 EU/mL [95% CI 195 to 293]), or ≥ 280 days (212 EU/mL [95% CI 121 to 372]) (**Table J in S1 Data**). There was a trend for increasing responses 21 days after MVA-BN-Filo vaccination as the interval lengthened. GMCs were 15,555 EU/mL (95% CI 10,907 to 22,184, *n =* 17) for 140 days, 14,995 EU/mL (95% CI 11,855 to 18,965, *n =* 52) for 196 days, 16,149 EU/mL (95% CI 13,882 to 18,786, *n =* 77) for 252 days, and 23,897 EU/mL (95% CI 16,703 to 34,188, *n* = 21) for intervals of ≥280 days (**Table J in S1 Data**).

#### Neutralising antibody responses against EBOV GP

**Fig 3:**
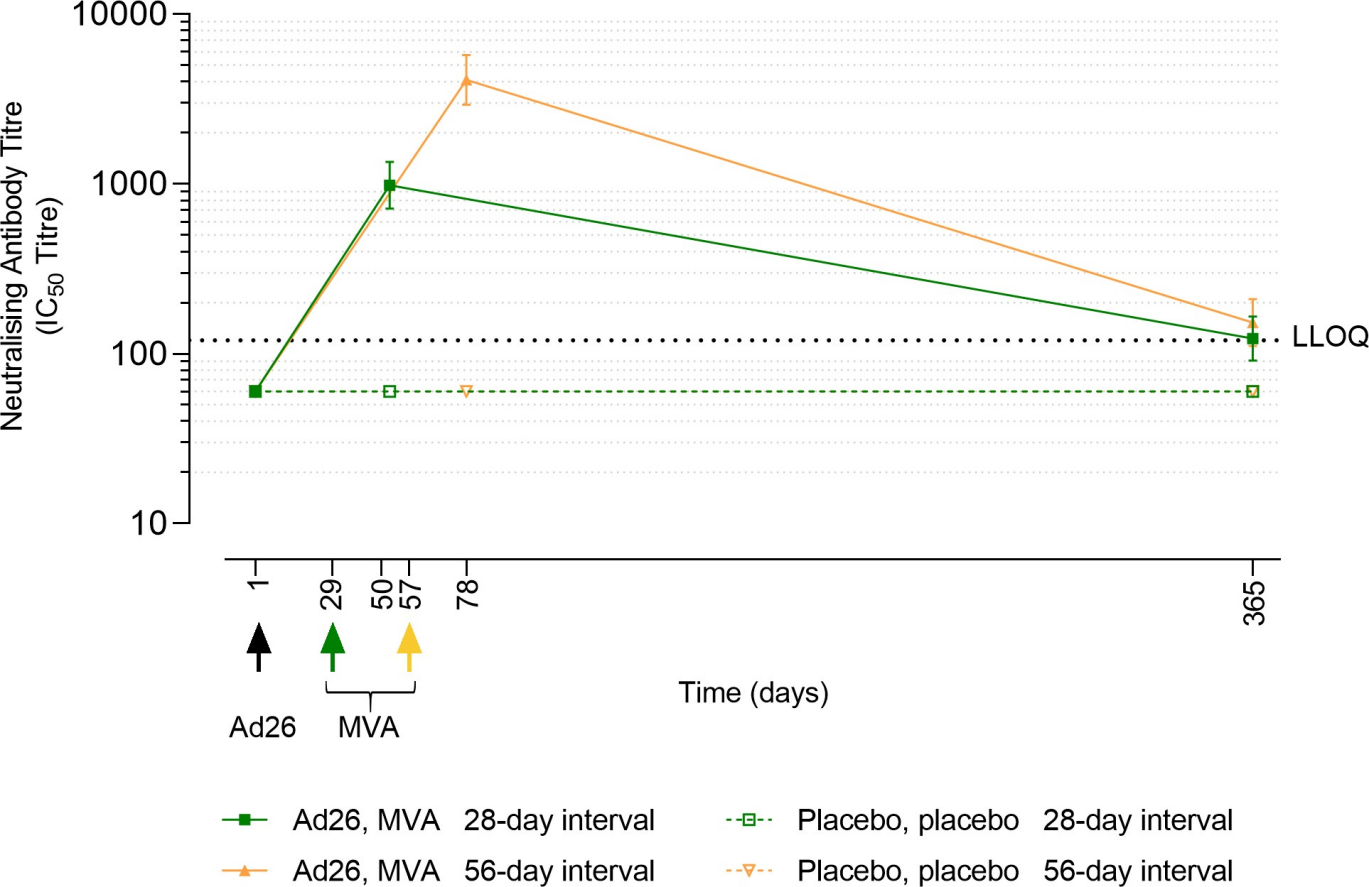
Anti-EBOV GP neutralising antibody responses in healthy adults. The error bars represent the GMT (IC_50_) and its 95% CI. Subset of healthy adults administered with Ad26.ZEBOV on Day 1 and MVA-BN-Filo 28 or 56 days later as indicated. Responses in placebo groups are shown as open symbols. Black dotted line represents the LLOQ. Vaccines: Ad26 = Ad26.ZEBOV at a dose of 5 × 10^10^ vp; MVA = MVA-BN-Filo at a dose of 1 × 10^8^ Inf.U. CI, confidence interval; GMT, geometric mean titre; IC_50_, half maximal inhibitory concentration; LLOQ, lower limit of quantification.

Neutralising antibodies against EBOV GP were tested in a subset of per-protocol analysis set healthy adult participants: 76 vaccinees and 21 placebo recipients (**Table K in S1 Data**). Neutralising antibodies were not observed in placebo recipients at any time point. At 21 days post-dose 2, 92% and 97% in the 28- and 56-day regimens, respectively, demonstrated neutralising antibodies against EBOV GP; geometric mean titres (GMTs) were 982 half maximal inhibitory concentration (IC_50_) (95% CI 714 to 1,350) and 4,100 IC_50_ (95% CI 2,927 to 5,745) (**Fig 3 and Table K in S1 Data**). Titres decreased by Day 365 with 21% and 24% of the tested 28- and 56-day group participants, respectively, still displaying neutralising activity; GMTs were 123 IC_50_ (95% CI <LLOQ–165) and 153 IC_50_ (95% CI <LLOQ–210). There were strong correlations between the neutralising and binding antibody responses 21 days post-dose 2 (Spearman coefficient = 0.735 in 28-day interval group and 0.852 in 56-day interval group) and at Day 365 (Spearman coefficient = 0.746 in 28-day interval group and 0.631 in 56-day interval group) (**S1 Fig**).

#### Neutralising antibody responses against Ad26 vector

Before vaccination, Ad26-specific neutralising antibodies were analysed in 98 healthy adults: 48 of the 28-day group (39 vaccinees, 9 placebo) and 50 of the 56-day group (38 vaccinees and 12 placebo). An overall Ad26-specific seroprevalence rate of 91% with a GMT of 106 IC_90_ (95% CI 79 to 142) was detected. Similarly, an overall 83% seroprevalence rate (GMT: 73 IC_90_) was observed for the HIV-infected adults (**Table L in S1 Data**). Potential impact of Ad26-specific preexisting neutralising antibodies on vaccine-induced EBOV GP–specific humoral immune responses was examined at a participant level via descriptive statistical correlation analyses (**S2 and S3 Figs**). Negligible correlations were observed between Ad26-specific neutralising antibody titres at baseline and EBOV GP–specific binding antibody concentrations 21 days post-dose 2 (Spearman correlation coefficients: −0.02 and 0.22 for 28- and 56-day intervals in healthy participants, respectively; 0.02 and 0.01 for 28- and 56-day intervals in HIV-infected participants, respectively), as well as EBOV GP–specific neutralising antibody titres 21 days post-dose 2 (Spearman correlation coefficients: −0.15 and 0.11 for 28- and 56-day intervals, respectively).

### Cellular immune responses against EBOV GP

Cellular immune responses were tested in a subset of healthy (16%) and HIV-infected (21%) participants. Twenty-one days post-dose 2, CD4+ T cell responses were observed in 32% (56-day interval) and 50% (28-day interval) of healthy participants. The median value tended to be numerically higher in participants vaccinated in the 28-day interval (0.11%) than the 56-day interval (0.06%) (**S4A Fig**). At the same time point, CD8+ T cell responses were detected in 29% (28-day interval) and 30% (56-day interval) of healthy participants (median value: 0.05%; <threshold, respectively) (**S4B Fig**).

In HIV-infected participants (56-day interval), 21 days post-dose 2, CD4+ T cell responses were observed in 40% of participants (median 0.18%). CD8+ T cell responses were observed in 17% of participants (median value <threshold) (**S5A and S5B Fig**). IFNγ ELISpot data (**Table M in S1 Data)** are described in **S5 Text**.

## Discussion

This Phase II study confirmed the safety, tolerability, and immunogenicity of heterologous 2-dose Ad26.ZEBOV, MVA-BN-Filo vaccination regimen against EBOV in African adults, including those with HIV infection. Safety and tolerability profiles were consistent with those observed in Phase I studies in European and African populations [15–17] and a Phase II study in a European population [22]. There were no deaths or SAEs associated with vaccination. Most AEs associated with either vaccine were transient and mild to moderate in severity, consisting mainly of injection-site pain, fatigue, headache, and myalgia.

As many conditions can influence the response to Ebola vaccines [23], it is important to ensure that local factors do not affect responses to vaccines. We previously reported the immune responses to the Ad26.ZEBOV, MVA-BN-Filo heterologous 2-dose regimen in Phase I studies in African populations [16,17]. The present study confirms these data and further demonstrates that HIV infection (well controlled by highly active antiretroviral therapy), a condition prevalent in African populations that could be expected to influence vaccine efficacy, did not have any apparent influence on those immune responses. The magnitude of the antibody responses 21 days post-dose 2 in our representative African population was similar to that observed in a Phase II study performed in Europe with a similar design, with comparable levels of EBOV GP–specific binding antibodies and virus neutralising activity 21 days post-dose 2 with 28- and 56-day vaccination intervals, measured in the same laboratory [22].

We have also reported safety and immunogenicity data of the 2-dose heterologous Ad26.ZEBOV and MVA-BN-Filo vaccination regimen, given 8 weeks apart, in a 2-stage trial in Sierra Leone. This study was initially foreseen as a safety and clinical efficacy study but was amended to become a safety and immunogenicity study after waning of the outbreak in Sierra Leone [24,25].

In addition, our study confirms the optimal interval between the 2 vaccinations, i.e., 56 days. Strong immune responses are observed with each of the 3 intervals tested, but increasing the interval from 28 to 56 days resulted in 2-fold higher levels of binding antibodies in healthy adults and 4-fold higher levels of neutralising antibodies. Prolonging the interval from 56 to 84 days did not further increase these immune responses, confirming the 56-day regimen as the optimal choice to achieve a high-response magnitude with a reasonably short vaccination schedule.

Some healthy adult participants either did not receive dose 2 or received dose 2 outside of their protocol-defined interval. However, sensitivity analyses of immune responses among participants who received their second vaccination “out-of-window” confirmed that extending the interval between Ad26.ZEBOV and MVA-BN-Filo vaccinations to >280 days resulted in immune responses that were at least as high in magnitude as the 56-day interval.

Although >90% of the adult participants had preexisting neutralising antibodies against the Ad26 vector, there was no indication that this had any impact on the vaccine-induced immune responses based on descriptive statistical analyses.

A mechanistic correlate of protection has not yet been determined, and there is no human correlate of protection against EBOV infection. As demonstrated in nonhuman primate lethal challenge models, EBOV GP–specific IgG binding antibodies correlated strongly with protection and, thus, can be considered as a measure of efficacy [26,27]. When measured from 10 days after a single dose of the rVSV-ZEBOV-GP vaccine in a ring vaccination strategy in the 2014 to 2016 and 2018 to 2020 outbreaks, protective efficacy was found to be 100% and 97.5%, respectively [7,8]. This efficacy was achieved with an rVSV-ZEBOV-GP vaccine formulation that has been reported to elicit a GMC of 1,262 EU/mL 28 days after vaccination in non-African populations [28]. This GMC was measured using the same assay in the same laboratory as our study, in which the Ad26.ZEBOV, MVA-BN-Filo vaccine regimen elicited EBOV GP–specific antibody concentrations from 3,085 to 7,518 EU/mL 21 days after the second vaccination in these African populations, depending on the interval between vaccinations.

One year post-Ad26.ZEBOV vaccination, binding antibodies were observed in 78% to 88% of participants. An Ad26.ZEBOV booster dose at 1 year elicited strong anamnestic responses with 55-fold increases in binding antibodies in both 28- and 56-day groups within 7 days of the booster dose. This observation suggests that the 2-dose Ad26.ZEBOV, MVA-BN-Filo vaccine regimen had established immune memory [29] that could rapidly be reactivated, which could be very important in the context of recurrent epidemics observed in Africa.

The numerous EBOV outbreaks in the DRC highlight the need for a prophylactic vaccine against this virulent disease. Current WHO SAGE recommendations are to use the single-dose rVSV-ZEBOV-GP vaccination for reactive use in those at high risk of contracting EBOV and prophylactic use and evaluation of the heterologous 2-dose Ad26.ZEBOV, MVA-BN-Filo regimen for those at less imminent risk [12]. Our study adds to the database of knowledge about this heterologous 2-dose strategy, particularly in the target population of African adults. The finding that increasing the interval between vaccinations from 56 to 84 days (and even further) resulted in similarly high binding antibody responses allows for potential flexibility in vaccinations, which might be useful in practice. The second part of our study in paediatric participants to ensure that the strategy is safe and immunologically effective in children as young as 4 years of age will be published separately. Additional studies are already underway to further evaluate the Ad26.ZEBOV, MVA-BN-Filo vaccination regimen in African populations, including younger children and infants, pregnant women, and healthcare workers who may have to travel to regions recurrently affected by outbreaks such as in the DRC.

Study limitations include that some healthy adult participants either did not receive dose 2 or received dose 2 outside of their protocol-defined interval. However, sensitivity analyses of immune responses among participants who received their second vaccination “out-of-window” showed that extending the interval between Ad26.ZEBOV and MVA-BN-Filo vaccinations to >280 days resulted in immune responses that were at least as high in magnitude as the 56-day interval. Another limitation is that the follow-up period was limited to 365 days for the majority of participants, and so it was not possible to determine whether immune responses persisted beyond this time period. However, this limitation could not be avoided as a follow-up period had to be determined before commencing the study, and, reassuringly, modelling results [30] suggest that immune responses persist beyond this time. Finally, no formal statistical testing of safety or immune response data was originally planned for this study. Although post hoc statistical comparisons were performed, no direct conclusions on the differences in safety and immunogenicity observed between regimens or between healthy and HIV-infected groups can be made; a clinically meaningful difference in terms of binding antibody levels is not known.

In conclusion, our study confirms that the heterologous 2-dose Ad26.ZEBOV, MVA-BN-Filo vaccination regimen has a safety, tolerability, and immunogenicity profile in African adults similar to that already demonstrated in European volunteers. These data supported the prophylactic indication against EBOV disease as licensed by the European Union.

## Supporting information

S1 CONSORT Checklist (DOCX)

S1 Study Protocol (PDF)

S1 Text Inclusion and exclusion criteria. (DOCX)

S2 Text Participant subset selection criteria for exploratory assays. (DOCX)

S3 Text Determination of neutralising antibody activity in an EBOV GP psVNA. EBOV GP, Ebola virus glycoprotein; psVNA, pseudovirion neutralisation assay.
(DOCX)

S4 Text Sample interpretation and responder definition for cellular immune assays. (DOCX)

S5 Text EBOV GP–specific IFNγ producing T cell responses (IFNγ ELISpot). EBOV GP, Ebola virus glycoprotein; ELISpot, enzyme-linked immunospot; IFNγ, interferon gamma.
(DOCX)

S1 Data Table A. SAEs in the full analysis set after each vaccination dose, n (%). Table B. Solicited AEs* in the full analysis set: Healthy adults stratified to 18–50 years and > 50 years age groups, n (%). Table C. Comparison (healthy adults versus HIV-infected adults) of solicited and unsolicited AEs in the full analysis set after each vaccination dose based on Fisher’s exact test. Table D. Summary of fever in the full analysis set after each vaccination dose, n (%). Table E. EBOV GP–specific binding antibodies in healthy adult participants in the vaccine groups stratified to 18–50 years and >50 years age groups—per-protocol analysis set. Table F. EBOV GP–specific binding antibodies in healthy adult participants in the active vaccine groups—per-protocol analysis set. Table G. Comparison of EBOV GP–specific binding antibodies in healthy adult and HIV-infected adults in the active vaccine groups—per-protocol analysis set. Table H. EBOV GP–specific binding antibodies in HIV-infected adults in the active vaccine groups—per-protocol analysis set. Table I. Comparison of EBOV GP–specific binding antibodies in healthy adult versus HIV-infected adult participants in the active vaccine groups—per-protocol analysis set. Table J. EBOV GP–specific binding antibodies in healthy adults receiving the second dose of active vaccine outside the protocol-defined window. Table K. GMT of EBOV GP–specific pseudovirion neutralising antibodies titres (IC_50_) and responder rates in healthy adult active vaccine groups 1 and 2—per-protocol analysis set. Table L. Ad26 neutralising antibodies (Ad26 VNA, IC_90_ titre) in healthy and HIV-infected adults—per-protocol analysis set. Table M. EBOV GP–specific IFNγ producing T cell responses (IFNγ ELISpot) in healthy and HIV-infected adults—per-protocol analysis set. Ad26 VNA, Ad26-specific virus neutralisation assay; AE, adverse event; EBOV GP, Ebola virus glycoprotein; ELISpot, enzyme-linked immunospot; GMT, geometric mean titre; IFNγ, interferon gamma; SAE, serious adverse event.
(DOCX)

S1 Fig Correlations between EBOV GP–specific binding and neutralising antibodies in participants assigned to an active regimen. (A) 21 days post-dose 2. (B) 364 days post-dose 2. EBOV GP, Ebola virus glycoprotein; LLOQ, lower limit of quantification; ULOQ, upper limit of quantification.
(DOCX)

S2 Fig Correlations between Ad26-specific neutralising antibody titres at baseline and EBOV GP–specific binding and neutralising antibodies 21 days post-dose 2 in healthy adults. (A) Anti-EBOV GP IgG ELISA at 21 days post-dose 2 by Ad26 neutralisation assay at baseline. (B) EBOV GP neutralisation assay at 21 days post-dose 2 by Ad26 neutralisation assay at baseline. Ad26 VNA, Ad26-specific virus neutralisation assay; EBOV GP, Ebola virus glycoprotein; ELISA, enzyme-linked immunosorbent assay; IgG, immunoglobulin G; LLOQ, lower limit of quantification; ULOQ, upper limit of quantification.
(DOCX)

S3 Fig Correlations between Ad26-specific neutralising antibody titres at baseline and EBOV GP–specific binding antibodies at 21 days post-dose 2 in HIV-infected adults by anti-EBOV GP IgG ELISA. Ad26 VNA, Ad26-specific virus neutralisation assay; EBOV GP, Ebola virus glycoprotein; ELISA, enzyme-linked immunosorbent assay; IgG, immunoglobulin G; LLOQ, lower limit of quantification; ULOQ, upper limit of quantification.
(DOCX)

S4 Fig **EBOV GP–specific CD4+ (A) and CD8+ (B) T cell cytokine responses in healthy adult participants.** EBOV GP, Ebola virus glycoprotein; n, number of participants with data at that time point; NA, not applicable.
(DOCX)

S5 Fig **EBOV GP–specific CD4+ (A) and CD8+ (B) T cell cytokine responses in HIV-infected adult participants.** EBOV GP, Ebola virus glycoprotein; n, number of participants with data at that time point; NA, not applicable.
(DOCX)

## Abbreviations

Ad26 VNA: Ad26-specific virus neutralisation assay
AE: adverse event
CI: confidence interval
DRC: Democratic Republic of the Congo
EBOV: Ebola virus
EBOV GP: Ebola virus glycoprotein
ELISA: enzyme-linked immunosorbent assay
EU/mL: ELISA units/mL
ELISpot: enzyme-linked immunospot
FANG: Filovirus Animal Non-Clinical Group
FDA: Food and Drug Administration
GMC: geometric mean concentration
GMT: geometric mean titre
GP: glycoprotein
IC_50_: half maximal inhibitory concentration
ICS: intracellular cytokine staining
IgG: immunoglobulin G
IWRS: interactive web response system
LLOQ: lower limit of quantification
psVNA: pseudovirion neutralisation assay
SAE: serious adverse event
SAGE: Strategic Advisory Group of Experts on Immunization
SD: standard deviation
URTI: upper respiratory tract infection
WHO: World Health Organization
