# Supplementary material for: Safety and immunogenicity of 2-dose heterologous Ad26.ZEBOV, MVA-BN-Filo Ebola vaccination in healthy and HIV-infected adults: A randomised, placebo-controlled Phase II clinical trial in Africa
Source: PLoS Med. 2021 Oct 29;18(10):e1003813. doi: 10.1371/journal.pmed.1003813 (PMC8555783; doi:10.1371/journal.pmed.1003813)
Supplement: S1 Text — (DOCX) [file pmed.1003813.s003.docx]

**S1 Text. Inclusion and exclusion criteria**

**Inclusion criteria – healthy adults:**

1. Signed an informed consent form indicating that he/she understands the purpose of, and procedures required for, the study and is willing to participate in the study.
2. Man or woman aged 18 (or the legal age of consent in the jurisdiction in which the study was taking place) to 70 years of age, inclusive at randomisation.
3. Healthy in the investigator’s clinical judgment based on medical history, electrocardiogram (ECG), physical examination, and vital signs assessments at screening.
4. Healthy based on clinical laboratory tests performed at screening. If the results of the tests were outside the institutional normal reference ranges, the subject could be included only if the investigator judged the abnormalities or deviations from normal to be clinically insignificant or appropriate and reasonable for the population under study.
5. Before randomisation (on Day 1), a sexually active woman had to be either:
   1. of childbearing potential and practicing a highly effective method of birth control consistent with local regulations regarding the use of birth control methods for subjects participating in clinical studies, beginning at least 28 days prior to vaccination, OR
   2. not of childbearing potential: postmenopausal, permanently sterilised, or otherwise be incapable of pregnancy.
6. Women of childbearing potential had to have a negative serum pregnancy test (β-human chorionic gonadotropin, β-hCG) at screening and a negative urine pregnancy test (β-hCG) immediately prior to each study vaccination.
7. Men who were sexually active with a woman of childbearing potential had to use condoms for sexual intercourse beginning prior to study enrolment, unless a vasectomy had been performed more than 1 year prior to screening.
8. Available and willing to participate for the duration of the study visits and follow-up.
9. Willing and able to comply with the protocol requirements, including the prohibitions and restrictions specified in the protocol.
10. Willing to provide verifiable identification.
11. Had a means to be contacted.
12. Passed a Test of Understanding.

**Additional inclusion criteria – HIV-infected adults:**

1. Man or woman aged 18 (or the legal age of consent in the jurisdiction in which the study was taking place) to 50 years of age, inclusive at randomisation.
2. Documented human immunodeficiency virus (HIV) infection for at least 6 months prior to screening.
3. On a stable regimen of highly active antiretroviral therapy (HAART), taking into account the following criteria:
   1. HAART was defined as potent anti-HIV treatment including a combination of at least three antiretroviral agents (low-dose ritonavir did not count as an antiretroviral agent) whose purpose was to reduce viral load to undetectable levels. Mono- or bi-therapy was not allowed.
   2. HAART was considered stable if the subject did not change his/her antiretrovirals within the last 4 consecutive weeks prior to the start of screening. Changes in formulations were allowed.
   3. A subject entering the study on HAART had to have a CD4+ cell count >350 cells/μL at screening. Two documented results from at least 4 weeks apart were preferred. Viral load assessments could be used to establish effectiveness of viral suppression.
   4. The subject was willing to continue HAART throughout the study as directed by his/her local physician.

4. Otherwise reasonably good medical condition (absence of acquired immunodeficiency syndrome [AIDS]-defining illnesses or clinically significant disease), diagnosed on the basis of physical examination, medical history, and the investigator’s clinical judgment

**Exclusion criteria – Healthy and HIV-infected adults:**

1. Had received any candidate Ebola vaccine.
2. Had been diagnosed with Ebola virus disease, or prior exposure to Ebola virus, including travel to epidemic Ebola areas less than 1 month prior to screening.
   1. Note: Participation of international volunteers to Ebola operations was allowed, but they had to comply with the prohibitions and restrictions as specified in the protocol.
3. Had received any experimental candidate Ad26- or MVA-based vaccine in the past. Receipt of any approved vaccinia/smallpox vaccine or Ad-based candidate vaccine other than Ad26 at any time prior to study entry was allowed.
4. Had a known allergy or history of anaphylaxis or other serious adverse reactions to vaccines or vaccine products (including any of the constituents of the study vaccines), including a known allergy to egg, egg products, and aminoglycosides.
5. Had an acute illness (excluding minor illnesses such as diarrhoea or mild upper respiratory tract infection) or body temperature ≥38.0ºC on Day 1. Subjects with such symptoms were excluded from enrolment at that time, but could be rescheduled for enrolment at a later date.
6. Had HIV type 1 or type 2 infection.
7. Were pregnant, breastfeeding, or planning to become pregnant while enrolled in the study or within at least 3 months after dose 1 vaccination, up to 1 month after dose 2 vaccination (whichever took longer), or within at least 3 months after the booster dose.
8. Had a significant condition or clinically significant findings during screening of medical history, ECG (subjects ≥18 years of age), physical examination, vital signs, or laboratory testing for which, in the opinion of the investigator, study participation would not be in the best interest of the subject (eg, compromise the safety or well-being) or that could prevent, limit, or confound the protocol-specified assessments.
9. Had a history of or underlying liver or renal insufficiency, or significant cardiac, vascular, pulmonary (eg, persistent asthma), gastrointestinal, endocrine, neurologic, hematologic, rheumatologic, psychiatric, or metabolic disturbances.
10. Had a history of malignancy other than squamous cell or basal cell skin cancer, unless there had been surgical excision that was considered cured. Subjects with malignancies who were being treated or were not surgical cures were excluded.
11. Had undergone major surgery (per the investigator’s judgment) within the 4 weeks prior to screening or had planned major surgery through the course of the study (from screening until completion of the study).
12. Were post-organ or stem cell transplant whether or not with chronic immunosuppressive therapy.
13. Had received any disallowed therapies as described in Section vaccination on Day 1 before the planned dose 1.
14. Had received an investigational drug or an investigational vaccine, or had used an invasive investigational medical device, within 3 months prior to screening, or were participating or planning to participate in another clinical study during the study.
    1. Note: Participation in an observational clinical study was allowed.
15. Had donated a unit of blood within 8 weeks before Day 1 or were planning to donate blood until 42 days after the last study vaccination.
16. Had received blood products or immunoglobulin within 3 months prior to screening and during participation in the study.
17. Were abusing or previously had abused alcohol, recreational or narcotic drugs, which in the investigator’s opinion would compromise the subject’s safety and/or compliance with study procedures.
18. Had a history of chronic urticaria (recurrent hives).
19. Were unable to communicate reliably with the investigator.
20. In the opinion of the investigator, were unlikely to adhere to the requirements of the study.

Were an employee of the investigator or study site, with direct involvement in the proposed study or other studies under the direction of that investigator or study site, or were a family member of the employees or the investigator.
