# Supplementary material for: Safety and immunogenicity of 2-dose heterologous Ad26.ZEBOV, MVA-BN-Filo Ebola vaccination in healthy and HIV-infected adults: A randomised, placebo-controlled Phase II clinical trial in Africa
Source: PLoS Med. 2021 Oct 29;18(10):e1003813. doi: 10.1371/journal.pmed.1003813 (PMC8555783; doi:10.1371/journal.pmed.1003813)
Supplement: S2 Text — (DOCX) [file pmed.1003813.s004.docx]

**S2 Text. Participant subset selection criteria for exploratory assays**

(Neutralising antibody, intracellular cytokine staining, and interferon-gamma enzyme-linked immunospot assays).

Ebola virus (EBOV) glycoprotein (GP)-specific neutralising antibody activity was done in a randomly selected subset of participants (19%) considered large enough to provide a representative characterisation of the immune response. Included were 50/268 (41 active and nine placebo; healthy adults cohort) participants who were assigned to the 28-day interval and 50/268 (38 active and 12 placebo; healthy adults cohort) who were assigned to the 56-day interval. The selection was done in order to ensure that all exploratory assays were done on the same set of participants, in a blinded manner, considering the number of samples available for testing, the absence of major protocol deviations that could influence immunogenicity and aiming for a proportional distribution across the countries.

IFN‑γ and/or IL-2 and/or TNF-α producing CD4+ and CD8+ T cells were determined in an intracellular cytokine staining (ICS) assay at baseline, 21 days post-dose 2, and 1 year post-dose 1 in a subset (16%) of healthy adult participants assigned to the 28‑day (n = 46/268; 37 active and nine placebo) and 56-day (n = 38/268; 31 active and seven placebo) interval schedules, and in a subset (21%) of human immunodeficiency virus (HIV)-infected adult participants assigned to the 56-day interval schedule (n = 15/71; 13 active and two placebo).

The IFN-γ producing T cell response was determined in an IFN‑γ ELISpot assay at baseline, 21 days post-dose 2, and 1 year post-dose 1 in a subset (16%) of healthy adult participants assigned to the 28‑day (n = 46/268; 37 active and nine placebo) and 56-day (n = 38/268; 31 active and seven placebo) interval schedules, and in a subset (21%) of HIV-infected adult participants assigned to the 56‑day interval schedule (n = 15/71; 13 active and two placebo).

For these analyses, a subset of participants was intended to be randomly selected following the criteria adopted for EBOV GP-specific neutralising antibody analyses reported above; however, due to the limited number of samples, all participants were selected based on the availability of their biological materials and absence of major protocol deviations that could influence immunogenicity.
