# Supplementary material for: Safety and immunogenicity of 2-dose heterologous Ad26.ZEBOV, MVA-BN-Filo Ebola vaccination in healthy and HIV-infected adults: A randomised, placebo-controlled Phase II clinical trial in Africa
Source: PLoS Med. 2021 Oct 29;18(10):e1003813. doi: 10.1371/journal.pmed.1003813 (PMC8555783; doi:10.1371/journal.pmed.1003813)
Supplement: S3 Text — EBOV GP, Ebola virus glycoprotein; psVNA, pseudovirion neutralisation assay. (DOCX) [file pmed.1003813.s005.docx]

**S3 Text. Determination of neutralising antibody activity in an EBOV GP pseudovirion neutralisation assay**

This assay to assess the functionality of vaccine-induced antibody responses was developed at Monogram Biosciences (San Francisco, CA, USA), and was qualified with human serum. Samples for the current report were assayed at Monogram Biosciences according to the standard operating procedure ‘Crucell Ebola virus (EBOV) Neutralization Assay’.

Pseudovirions expressing the glycoprotein of an EBOV isolate from the 2014 outbreak (Makona variant) were produced in human embryonic kidney 293 (HEK293) cells by transfection with a GP expression plasmid and a vector encoding a firefly luciferase gene and all of the human immunodeficiency virus (HIV) type 1 genes required for viral replication, except for the envelope gene. Sera were pretreated to remove any nonspecific neutralising factors. A fixed amount of pseudovirions was mixed with a series of serial- dilutions of serum samples. Following incubation, the samples were transferred to a HEK293 cell monolayer. The inhibition of pseudovirion infection was measured by luciferase reporter gene expression. The assay responses of the serially diluted samples were plotted in a 4-parameter logistic regression curve and the 50% inhibitory concentration (IC_50_) of each curve was reported as each a neutralisation titre for each serum sample.

A psVNA result (IC_50_ titre) was considered positive if the specific IC_50_ titre was more than three times amphotropic murine leukaemia virus (aMLV) and above the assay-specific lower limit of quantification (LLOQ). Values that were less than three times aMLV or below the LLOQ were imputed with LLOQ/2 (120/2). For the calculation of fold increases, values that were less than three times aMLV or below the LLOQ were imputed with the LLOQ. The psVNA values were log_10_-transformed before further handling. The log_10_-transformed values were used throughout the entire analysis.

psVNA titres are expressed as geometric mean titres (GMT) with 95% confidence intervals (CI) and group proportions of responders at each timepoint. psVNA responders were defined as negative at baseline and positive post vaccination with a titre two-fold higher than LLOQ (120 IC_50_ titre), or positive at baseline with greater than two-fold increase post vaccination.
