# Supplementary material for: Safety and immunogenicity of 2-dose heterologous Ad26.ZEBOV, MVA-BN-Filo Ebola vaccination in healthy and HIV-infected adults: A randomised, placebo-controlled Phase II clinical trial in Africa
Source: PLoS Med. 2021 Oct 29;18(10):e1003813. doi: 10.1371/journal.pmed.1003813 (PMC8555783; doi:10.1371/journal.pmed.1003813)
Supplement: S4 Text — (DOCX) [file pmed.1003813.s006.docx]

**S4 Text. Sample interpretation and responder definition for cellular immune assays**

**Intracellular cytokine staining (ICS)**

For each antigen, the total cytokine count of the considered antigen (IFN-γ+ or IL-2+ or TNF-α+) was compared with the total cytokine count in all negative control samples with Fisher’s exact test. The test was repeated for both peptide pools (GP1 and GP2) and for each antigen. If the observed p-value for at least one peptide pool (GP1 or GP2) was below 10 ^-5^/2 (division by 2 due to Bonferroni correction as this is done for each pool), the sample interpretation was considered positive for that antigen.

A participant was a responder at a considered timepoint if sample interpretation was negative at baseline but positive post-baseline and the post-baseline value was greater than 2x Sponsor-defined assay-specific threshold (0.04%), or sample interpretation was positive at both baseline and post-baseline and there was a greater than two-fold increase from baseline in background adjusted total cytokine response. Values below the assay-specific threshold were imputed with threshold /2 (0.02%). For the calculation of fold increases, values below the assay-specific threshold were imputed with the threshold.

**ELISpot for interferon (IFN)-γ producing T cell responses to EBOV GP**

For IFN-γ ELISpot, sample interpretation was determined for each Ebola virus (EBOV) GP peptide pool (GP1 and GP2) separately. If a sample was positive for at least one of the peptide pools, the sample was considered positive. Overall, a result was considered positive if the EBOV peptide pool-stimulated readout was greater than three-fold the unstimulated readout and the unstimulated-subtracted value was greater than the threshold (50 spot forming units [SFU]/10^6^ PBMC).

Values below the positivity threshold were imputed with half of the threshold. For the calculation of fold increases, values below the positivity threshold were imputed with the threshold.

A participant was a responder at a considered timepoint if the sample interpretation was negative at baseline and positive post-baseline and the post-baseline value was greater than 2x the threshold, or if the sample interpretation was positive both at baseline and post-baseline and there was a greater than two-fold increase from baseline.
