# Supplementary material for: Safety and immunogenicity of 2-dose heterologous Ad26.ZEBOV, MVA-BN-Filo Ebola vaccination in healthy and HIV-infected adults: A randomised, placebo-controlled Phase II clinical trial in Africa
Source: PLoS Med. 2021 Oct 29;18(10):e1003813. doi: 10.1371/journal.pmed.1003813 (PMC8555783; doi:10.1371/journal.pmed.1003813)
Supplement: S5 Text — EBOV GP, Ebola virus glycoprotein; ELISpot, enzyme-linked immunospot; IFNγ, interferon gamma. (DOCX) [file pmed.1003813.s007.docx]

**S5 Text. EBOV GP-specific IFN**-**γ producing T cell responses (IFN-γ ELISpot)**

IFN-γ T cell responses were detected in 27% of healthy adult participants 21 days post-dose 2 (median range: 61–73 SFU/10^6^PBMC). No apparent differences between the 28- and 56-day intervals were observed.

At the same timepoint, IFN-γ T cell responses were detected in 17% of human immunodeficiency virus (HIV)-infected participants who received Ad26.ZEBOV, MVA-BN-Filo in a 56-day interval (median: <LLOQ).

Median values and responder rates for both healthy and HIV-infected cohorts are displayed in **Table J in S1 Data.**
