# Supplementary material for: Safety and immunogenicity of 2-dose heterologous Ad26.ZEBOV, MVA-BN-Filo Ebola vaccination in healthy and HIV-infected adults: A randomised, placebo-controlled Phase II clinical trial in Africa
Source: PLoS Med. 2021 Oct 29;18(10):e1003813. doi: 10.1371/journal.pmed.1003813 (PMC8555783; doi:10.1371/journal.pmed.1003813)
Supplement: S1 Data — Table A. SAEs in the full analysis set after each vaccination dose, n (%). Table B. Solicited AEs* in the full analysis set: Healthy adults stratified to 18–50 years and > 50 years age groups, n (%). Table C. Comparison (healthy adults versus HIV-infected adults) of solicited and unsolicited AEs in the full analysis set after each vaccination dose based on Fisher’s exact test. Table D. Summary of fever in the full analysis set after each vaccination dose, n (%). Table E. EBOV GP–specific binding antibodies in healthy adult participants in the vaccine groups stratified to 18–50 years and >50 years age groups—per-protocol analysis set. Table F. EBOV GP–specific binding antibodies in healthy adult participants in the active vaccine groups—per-protocol analysis set. Table G. Comparison of EBOV GP–specific binding antibodies in healthy adult and HIV-infected adults in the active vaccine groups—per-protocol analysis set. Table H. EBOV GP–specific binding antibodies in HIV-infected adults in the active vaccine groups—per-protocol analysis set. Table I. Comparison of EBOV GP–specific binding antibodies in healthy adult versus HIV-infected adult participants in the active vaccine groups—per-protocol analysis set. Table J. EBOV GP–specific binding antibodies in healthy adults receiving the second dose of active vaccine outside the protocol-defined window. Table K. GMT of EBOV GP–specific pseudovirion neutralising antibodies titres (IC50) and responder rates in healthy adult active vaccine groups 1 and 2—per-protocol analysis set. Table L. Ad26 neutralising antibodies (Ad26 VNA, IC90 titre) in healthy and HIV-infected adults—per-protocol analysis set. Table M. EBOV GP–specific IFNγ producing T cell responses (IFNγ ELISpot) in healthy and HIV-infected adults—per-protocol analysis set. Ad26 VNA, Ad26-specific virus neutralisation assay; AE, adverse event; EBOV GP, Ebola virus glycoprotein; ELISpot, enzyme-linked immunospot; GMT, geometric mean titre; IFNγ, interferon gamma; SAE, [file pmed.1003813.s008.docx]

| **Table A.** **SAEs in the full analysis set after each vaccination dose, n (%).** | | | | | | | | | | | |
| --- | --- | --- | --- | --- | --- | --- | --- | --- | --- | --- | --- |
|  | | **Healthy adults** | | | | | | **HIV-infected adults** | | | |
|  | | Group 1:  28 day-interval | | Group 2:  56 day-interval | | Group 3:  84 day-interval | | Group 1:  28 day-interval | | Group 2:  56 day-interval | |
|  | | **Ad26** | **Placebo** | **Ad26** | **Placebo** | **Ad26** | **Placebo** | **Ad26** | **Placebo** | **Ad26** | **Placebo** |
| **Entire study** | N | 225 | 43 | 224 | 44 | 110 | 22 | 59 | 12 | 59 | 12 |
| Any event, n (%) |  | 7 (3.1) | 1 (2.3) | 7 (3.1) | 0 | 6 (5.5) | 0 | 1 (1.7) | 0 | 1 (1.7) | 0 |
| **Post-dose 1** | N | 225 | 43 | 224 | 44 | 110 | 22 | 59 | 12 | 59 | 12 |
| Any event, n (%) | | 1 (0.4) | 0 | 0 | 0 | 0 | 0 | 0 | 0 | 0 | 0 |
| Infections and infestations | | 1 (0.4) | 0 | 0 | 0 | 0 | 0 | 0 | 0 | 0 | 0 |
| Cellulitis | | 1 (0.4) | 0 | 0 | 0 | 0 | 0 | 0 | 0 | 0 | 0 |
|  | | **Ad26** | **Placebo** | **Ad26** | **Placebo** | **Ad26** | **Placebo** | **Ad26** | **Placebo** | **Ad26** | **Placebo** |
| **Post-dose 1 follow-up** | N | 85 | 16 | 224 | 44 | 110 | 22 | 11 | 0 | 59 | 12 |
| Any event, n (%) | | 1 (1.2) | 0 | 3 (1.3) | 0 | 1 (0.9) | 0 | 0 | 0 | 0 | 0 |
| Gastrointestinal disorders | | 1 (1.2) | 0 | 1 (0.4) | 0 | 0 | 0 | 0 | 0 | 0 | 0 |
| Inguinal hernia | | 1 (1.2) | 0 | 0 | 0 | 0 | 0 | 0 | 0 | 0 | 0 |
| Obstruction gastric | | 0 | 0 | 1 (0.4) | 0 | 0 | 0 | 0 | 0 | 0 | 0 |
| Infections and infestations | | 0 | 0 | 1 (0.4) | 0 | 1 (0.9) | 0 | 0 | 0 | 0 | 0 |
| Malaria | | 0 | 0 | 1 (0.4) | 0 | 1 (0.9) | 0 | 0 | 0 | 0 | 0 |
| Metabolism and nutrition disorders | | 0 | 0 | 1 (0.4) | 0 | 0 | 0 | 0 | 0 | 0 | 0 |
| Electrolyte imbalance | | 0 | 0 | 1 (0.4) | 0 | 0 | 0 | 0 | 0 | 0 | 0 |
| Pregnancy, puerperium, and perinatal conditions | | 0 | 0 | 1 (0.4) | 0 | 0 | 0 | 0 | 0 | 0 | 0 |
| Abortion spontaneous | | 0 | 0 | 1 (0.4) | 0 | 0 | 0 | 0 | 0 | 0 | 0 |
| Renal and urinary disorders | | 0 | 0 | 1 (0.4) | 0 | 0 | 0 | 0 | 0 | 0 | 0 |
| Acute kidney injury | | 0 | 0 | 1 (0.4) | 0 | 0 | 0 | 0 | 0 | 0 | 0 |
|  | | **MVA** | **Placebo** | **MVA** | **Placebo** | **MVA** | **Placebo** | **MVA** | **Placebo** | **MVA** | **Placebo** |
| **Post-dose 2** | N | 219 | 39 | 200 | 39 | 98 | 21 | 58 | 12 | 59 | 12 |
| Any event, n (%) | | 0 | 1 (2.6) | 1 (0.5) | 0 | 0 | 0 | 0 | 0 | 0 | 0 |
| Ear and labyrinth disorders | | 0 | 1 (2.6) | 0 | 0 | 0 | 0 | 0 | 0 | 0 | 0 |
| Meniere's disease | | 0 | 1 (2.6) | 0 | 0 | 0 | 0 | 0 | 0 | 0 | 0 |
| Eye disorders | | 0 | 0 | 1 (0.5) | 0 | 0 | 0 | 0 | 0 | 0 | 0 |
| Cataract | | 0 | 0 | 1 (0.5) | 0 | 0 | 0 | 0 | 0 | 0 | 0 |
|  | | **MVA** | **Placebo** | **MVA** | **Placebo** | **MVA** | **Placebo** | **MVA** | **Placebo** | **MVA** | **Placebo** |
| **Post-dose 2 follow-up** | N | 219 | 39 | 200 | 39 | 98 | 21 | 58 | 12 | 59 | 12 |
| Any event, n (%) | | 5 (2.3) | 1 (2.6) | 3 (1.5) | 0 | 5 (5.1) | 0 | 1 (1.7) | 0 | 1 (1.7) | 0 |
| Infections and infestations | | 1 (0.5) | 1 (2.6) | 2 (1.0) | 0 | 3 (3.1) | 0 | 0 | 0 | 0 | 0 |
| Malaria | | 1 (0.5) | 1 (2.6) | 2 (1.0) | 0 | 2 (2.0) | 0 | 0 | 0 | 0 | 0 |
| Pulmonary tuberculosis | | 0 | 0 | 0 | 0 | 1 (1.0) | 0 | 0 | 0 | 0 | 0 |
| Gastrointestinal disorders | | 0 | 0 | 1 (0.5) | 0 | 1 (1.0) | 0 | 0 | 0 | 0 | 0 |
| Inguinal hernia | | 0 | 0 | 1 (0.5) | 0 | 1 (1.0) | 0 | 0 | 0 | 0 | 0 |
| Pregnancy, puerperium, and perinatal conditions | | 1 (0.5) | 0 | 0 | 0 | 1 (1.0) | 0 | 0 | 0 | 0 | 0 |
| Abortion spontaneous | | 1 (0.5) | 0 | 0 | 0 | 0 | 0 | 0 | 0 | 0 | 0 |
| Anembryonic gestation | | 0 | 0 | 0 | 0 | 1 (1.0) | 0 | 0 | 0 | 0 | 0 |
| Congenital, familial, and genetic disorders | | 1 (0.5) | 0 | 0 | 0 | 0 | 0 | 0 | 0 | 0 | 0 |
| Dolichocolon | | 1 (0.5) | 0 | 0 | 0 | 0 | 0 | 0 | 0 | 0 | 0 |
| Eye disorders | | 1 (0.5) | 0 | 0 | 0 | 0 | 0 | 0 | 0 | 0 | 0 |
| Glaucoma | | 1 (0.5) | 0 | 0 | 0 | 0 | 0 | 0 | 0 | 0 | 0 |
| Injury, poisoning, and procedural complications | | 1 (0.5) | 0 | 0 | 0 | 0 | 0 | 1 (1.7)^a^ | 0 | 0 | 0 |
| Ligament sprain | | 1 (0.5) | 0 | 0 | 0 | 0 | 0 | 0 | 0 | 0 | 0 |
| Alcohol poisoning | | 0 | 0 | 0 | 0 | 0 | 0 | 1 (1.7)^a^ | 0 | 0 | 0 |
| Respiratory, thoracic, and mediastinal disorders | | 0 | 0 | 0 | 0 | 0 | 0 | 0 | 0 | 1 (1.7) | 0 |
| Dyspnoea | | 0 | 0 | 0 | 0 | 0 | 0 | 0 | 0 | 1 (1.7) | 0 |
|  | | | | | | | |  |  |  |  |
|  | | **Ad26** | **Placebo** | **Ad26** | **Placebo** |  |  |  |  |  |  |
| **Post-booster** | N | 34 | 8 | 39 | 9 | - | - | - | - | - | - |
| Any event, n (%) | | 0 | 0 | 0 | 0 |  |  |  |  |  |  |
|  | | | | | | | |  |  |  |  |
|  | | **Ad26** | **Placebo** | **Ad26** | **Placebo** |  |  |  |  |  |  |
| **Post-booster follow-up** | N | 34 | 8 | 39 | 9 | - | - | - | - | - | - |
| Any event, n (%) | | 0 | 0 | 0 | 0 |  |  |  |  |  |  |

Adverse events are coded using MedDRA version 21.1.
N, number of participants with data at that timepoint; n (%): number (percentage) of participants with one or more events, where the denominator is the number of participants with available reactogenicity data after the given dose; SAE, serious adverse event.

In healthy adults, in the 56-day Ad26, MVA interval regimen, one serious adverse event was reported during the screening phase (hepatitis C); this event is not counted in this table.

^a^Fatal case.

Ad26: Ad26.ZEBOV at a dose of 5x10^10^ vp; MVA: MVA-BN-Filo at a dose of 1x10^8^ Inf.U.

| **Table B. Solicited adverse events* in the full analysis set: Healthy adults stratified to 18–50 years and > 50 years age groups, n (%).** | | | | | | | | | | | | | |
| --- | --- | --- | --- | --- | --- | --- | --- | --- | --- | --- | --- | --- | --- |
|  | | **Age group: 18–50 years** | | | | | | **Age group: 51–70 Years** | | | | | |
|  | | Group 1:  28 day-interval | | Group 2:  56 day-interval | | Group 3:  84 day-interval | | Group 1:  28 day-interval | | Group 2:  56 day-interval | | Group 3:  84 day-interval | |
|  | | **Ad26** | **Placebo** | **Ad26** | **Placebo** | **Ad26** | **Placebo** | **Ad26** | **Placebo** | **Ad26** | **Placebo** | **Ad26** | **Placebo** |
| **Post-dose 1** | N | 195 | 38 | 195 | 39 | 98 | 20 | 30 | 5 | 29 | 5 | 12 | 2 |
| Solicited AE, n (%) | | 140 (71.8) | 27 (71.1) | 139 (71.3) | 27 (69.2) | 75 (76.5) | 14 (70.0) | 21 (70.0) | 3 (60.0) | 22 (75.9) | 4 (80.0) | 8 (66.7) | 1 (50.0) |
| Grade 3^†^ | | 4 (2.1) | 0 | 6 (3.1) | 1 (2.6) | 5 (5.1) | 1 (5.0) | 0 | 0 | 0 | 0 | 0 | 0 |
| Solicited local AE | | 109 (55.9) | 13 (34.2) | 103 (52.8) | 17 (43.6) | 57 (58.2) | 10 (50) | 14 (46.7) | 3 (60.0) | 18 (62.1) | 3 (60.0) | 6 (50.0) | 1 (50.0) |
| Grade 3^†^ | | 0 | 0 | 1 (0.5) | 0 | 1 (1.0) | 0 | 0 | 0 | 0 | 0 | 0 | 0 |
| Solicited systemic AE | | 127 (65.1) | 24 (63.2) | 120 (61.5) | 23 (59) | 68 (69.4) | 13 (65.0) | 19 (63.3) | 3 (60.0) | 20 (69.0) | 3 (60.0) | 7 (58.3) | 1 (50.0) |
| Grade 3^†^ | | 4 (2.1) | 0 | 6 (3.1) | 1 (2.6) | 5 (5.1) | 1 (5.0) | 0 | 0 | 0 | 0 | 0 | 0 |
|  | | **MVA** | **Placebo** | **MVA** | **Placebo** | **MVA** | **Placebo** | **MVA** | **Placebo** | **MVA** | **Placebo** | **MVA** | **Placebo** |
| **Post-dose 2** | N | 189 | 35 | 172 | 35 | 86 | 19 | 30 | 4 | 28 | 4 | 12 | 2 |
| Solicited AE, n (%) | | 133 (70.4) | 14 (40.0) | 124 (72.1) | 21 (60.0) | 65 (75.6) | 13 (68.4) | 19 (63.3) | 1 (25.0) | 24 (85.7) | 4 (100.0) | 8 (66.7) | 2 (100.0) |
| Grade 3^†^ | | 5 (2.6) | 1 (2.9) | 2 (1.2) | 1 (2.9) | 6 (7.0) | 0 | 0 | 1 (25.0) | 0 | 0 | 0 | 0 |
| Solicited local AE | | 110 (58.2) | 8 (22.9) | 93 (54.1) | 14 (40.0) | 53 (61.6) | 7 (36.8) | 16 (53.3) | 1 (25.0) | 20 (71.4) | 2 (50.0) | 4 (33.3) | 2 (100.0) |
| Grade 3^†^ | | 2 (1.1) | 0 | 0 | 0 | 2 (2.3) | 0 | 0 | 0 | 0 | 0 | 0 | 0 |
| Solicited systemic AE | | 104 (55.0) | 13 (37.1) | 103 (59.9) | 18 (51.4) | 57 (66.3) | 13 (68.4) | 17 (56.7) | 1 (25.0) | 18 (64.3) | 3 (75.0) | 7 (58.3) | 1 (50.0) |
| Grade 3^†^ | | 4 (2.1) | 1 (2.9) | 2 (1.2) | 1 (2.9) | 5 (5.8) | 0 | 0 | 1 (25.0) | 0 | 0 | 0 | 0 |
|  | | **Ad26** | **Placebo** | **Ad26** | **Placebo** | - | - | **Ad26** | **Placebo** | **Ad26** | **Placebo** | - | - |
| **Post-booster** | N | 28 | 7 | 28 | 9 | - | - | 6 | 1 | 11 | 0 | - | - |
| Solicited AE, n (%) | | 14 (50.0) | 3 (42.9) | 13 (46.4) | 4 (44.4) |  |  | 6 (100.0) | 0 | 9 (81.8) |  |  |  |
| Grade 3^†^ | | 0 | 0 | 1 (3.6) | 0 |  |  | 0 | 0 | 0 |  |  |  |
| Solicited local AE | | 12 (42.9) | 1 (14.3) | 9 (32.1) | 3 (33.3) |  |  | 6 (100.0) | 0 | 7 (63.6) |  |  |  |
| Grade 3^†^ | | 0 | 0 | 0 | 0 |  |  | 0 | 0 | 0 |  |  |  |
| Solicited systemic AE | | 11 (39.3) | 2 (28.6) | 11 (39.3) | 4 (44.4) |  |  | 6 (100.0) | 0 | 7 (63.6) |  |  |  |
| Grade 3^†^ | | 0 | 0 | 1 (3.6) | 0 |  |  | 0 | 0 | 0 |  |  |  |

*Solicited local and systemic AEs were recorded on diary cards by participants for seven days and unsolicited AEs until Day 28 after each vaccination. Solicited local AEs included pain, erythema, swelling, and pruritis; systemic AEs included nausea/vomiting, fatigue/malaise, headache, myalgia, arthralgia, and chills.

^†^Grade 3 was defined as symptoms causing inability to perform usual social and functional activities.

Vaccines: Ad26 = Ad26.ZEBOV at a dose of 5x10^10^ vp; MVA = MVA-BN-Filo at a dose of 1x10^8^ Inf.U.

AE, adverse event ; N, number of participants with data at that timepoint; n (%): number (percentage) of participants with one or more events, where the denominator is the number of participants with available reactogenicity data after the given dose.

**Table C. Comparison (healthy adults versus HIV-infected adults) of solicited and unsolicited adverse events in the full analysis set after each vaccination dose based on Fisher’s exact test.**

|  | | Healthy adults | HIV-infected adults | *p*-value* |
| --- | --- | --- | --- | --- |
|  | | **Ad26** | **Ad26** |  |
| **Post-dose 1** | **N** | 559 | 118 |  |
| Solicited local AE, n (%) | | 307 (54.9) | 69 (58.5) | 0.54 |
| Solicited systemic AE, n (%) | | 361 (64.6) | 80 (67.8) | 0.53 |
| Any fever, n (%) | | 25 (4.5) | 13 (11.0) | 0.01 |
| Any unsolicited AE, n (%) | | 201 (36) | 50 (42.4) | 0.21 |
|  | | **MVA** | **MVA** |  |
| **Post-dose 2** | **N** | 517 | 117 |  |
|  | |  |  |  |
| Solicited local AE, n (%) | | 296 (57.3) | 51 (43.6) | 0.01 |
| Solicited systemic AE, n (%) | | 306 (59.2) | 58 (49.6) | 0.06 |
| Any fever, n (%) | | 33 (6.4) | 3 (2.6) | 0.12 |
| Any unsolicited AE, n (%) | | 166 (32.1) | 44 (37.6) | 0.28 |
|  | |  |  |  |
| **Entire study** | **N** | 559 | 118 |  |
| Any SAE, n (%) | | 20 (3.6) | 2 (1.7) | 0.40 |
| *p-values based on Fisher’s exact test for comparing healthy adults against HIV-infected adults.  Vaccines: Ad26 = Ad26.ZEBOV at a dose of 5x10^10^ vp; MVA = MVA-BN-Filo at a dose of 1x10^8^ Inf.U.  AE, adverse event; N, number of participants with data at that timepoint; n (%): number (percentage) of participants with one or more events, where the denominator is the number of participants with available reactogenicity data after the given dose. | | | | |

| **Table D. Summary of fever in the full analysis set after each vaccination dose, n (%)** | | | | | | | | | | | | | | | | |
| --- | --- | --- | --- | --- | --- | --- | --- | --- | --- | --- | --- | --- | --- | --- | --- | --- |
|  | | **Healthy adults** | | | | | | | | **HIV-infected adults** | | | | | |  |
|  |  | Group 1:  28 day-interval | | Group 2:  56 day-interval | | Group 3:  84 day-interval | | All groups | | Group 1:  28 day-interval | | Group 2:  56 day-interval | | All groups | |  |
|  |  | **Ad26** | **Placebo** | **Ad26** | **Placebo** | **Ad26** | **Placebo** | **Ad26** | **Placebo** | **Ad26** | **Placebo** | **Ad26** | **Placebo** | **Ad26** | **Placebo** |  |
| **Post-dose 1** | N | 225 | 43 | 224 | 44 | 110 | 22 | 559 | 109 | 59 | 12 | 59 | 12 | 118 | 24 |  |
| Fever (any), n (%) |  | 10 (4.4) | 1 (2.3) | 13 (5.8) | 3 (6.8) | 2 (1.8) | 0 | 25 (4.5) | 4 (3.6) | 5 (8.5) | 2 (16.7) | 8 (13.6) | 0 | 13 (11.0) | 2 (8.3) |  |
| Grade 1 |  | 6 (2.7) | 0 | 8 (3.6) | 2 (4.5) | 2 (1.8) | 0 | 16 (2.8) | 2 (1.8) | 1 (1.7) | 2 (16.7) | 4 (6.8) | 0 | 5 (4.2) | 2 (8.3) |  |
| Grade 2 |  | 3 (1.3) | 1 (2.3) | 2 (0.9) | 1 (2.3) | 0 | 0 | 5 (0.9) | 2 (1.8) | 3 (5.1) | 0 | 2 (3.4) | 0 | 5 (4.2) | 0 |  |
| Grade 3 |  | 1 (0.4) | 0 | 3 (1.3) | 0 | 0 | 0 | 4 (0.7) | 0 | 1 (1.7) | 0 | 2 (3.4) | 0 | 3 (2.5) | 0 |  |
|  |  | **MVA** | **Placebo** | **MVA** | **Placebo** | **MVA** | **Placebo** | **MVA** | **Placebo** | **MVA** | **Placebo** | **MVA** | **Placebo** | **MVA** | **Placebo** |  |
| **Post-dose 2** | N | 219 | 39 | 200 | 39 | 98 | 21 | 517 | 99 | 58 | 12 | 59 | 12 | 117 | 24 |  |
| Fever (any), n (%) |  | 13 (5.9) | 1 (2.6) | 11 (5.5) | 2 (5.1) | 9 (9.2) | 0 | 33 (6.4) | 3 (3.0) | 2 (3.4) | 1 (8.3) | 1 (1.7) | 2 (16.7) | 3 (2.6) | 3 (12.5) |  |
| Grade 1 |  | 8 (3.7) | 0 | 5 (2.5) | 1 (2.6) | 2 (2.0) | 0 | 15 (2.9) | 1 (1.0) | 1 (1.7) | 0 | 1 (1.7) | 1 (8.3) | 2 (1.7) | 1 (4.2) |  |
| Grade 2 |  | 2 (0.9) | 0 | 5 (2.5) | 1 (2.6) | 2 (2.0) | 0 | 9 (1.7) | 1 (1.0) | 1 (1.7) | 1 (8.3) | 0 | 0 | 1 (0.8) | 1 (4.2) |  |
| Grade 3 |  | 3 (1.4) | 1 (2.6) | 1 (0.5) | 0 | 5 (5.1) | 0 | 9 (1.7) | 1 (1.0) | 0 | 0 | 0 | 1 (8.3) | 0 | 1 (4.2) |  |
|  |  | **Ad26** | **Placebo** | **Ad26** | **Placebo** |  |  | **Ad26** | **Placebo** |  |  |  |  |  |  |  |
| **Post-booster** | N | 34 | 8 | 39 | 9 | NA | NA | 73 | 17 |  |  |  |  |  |  |  |
| Fever (any), n (%) |  | 1 (2.9) | 0 | 3 (7.7) | 0 | NA | NA | 4 (5.5) | 0 |  |  |  |  |  |  |  |
| Grade 1 |  | 0 | 0 | 1 (2.6) | 0 | NA | NA | 1 (1.4) | 0 |  |  |  |  |  |  |  |
| Grade 2 |  | 1 (2.9) | 0 | 1 (2.6) | 0 | NA | NA | 2 (2.7) | 0 |  |  |  |  |  |  |  |
| Grade 3 |  | 0 | 0 | 1 (2.6) | 0 | NA | NA | 1 (1.4) | 0 |  |  |  |  |  |  |  |

Vaccines: Ad26 = Ad26.ZEBOV at a dose of 5x10^10^ vp; MVA = MVA-BN-Filo at a dose of 1x10^8^ Inf.U.

HIV, human immunodeficiency virus; Inf.U, Infectious Units; N, number of participants with data at that timepoint; n (%): number (percentage) of participants with one or more events, where the denominator is the number of participants with available reactogenicity data after the given dose; NA, not applicable, vp, viral particles.

| **Table E. EBOV-GP-specific binding antibodies in healthy adult participants in the vaccine groups stratified to 18–50 years and > 50 years age groups – per-protocol analysis set.** | | | | | | | | | | | | | | | | | | |
| --- | --- | --- | --- | --- | --- | --- | --- | --- | --- | --- | --- | --- | --- | --- | --- | --- | --- | --- |
|  | **Age group: 18–50 years** | | | | | | **Age group: 51–70 Years** | | | | | | | | | | | |
|  | Group 1:  28 day-interval | | Group 2:  56 day-interval | | Group 3:  84 day-interval | | Group 1:  28 day-interval | | | | Group 2:  56 day-interval | | | | Group 3:  84 day-interval | | | |
|  | **Ad26, MVA** | **Placebo, Placebo** | **Ad26, MVA** | **Placebo, Placebo** | **Ad26, MVA** | **Placebo, Placebo** | **Ad26, MVA** | | **Placebo, Placebo** | | **Ad26, MVA** | | **Placebo, Placebo** | | **Ad26, MVA** | | **Placebo, Placebo** | |
| Day 1 (Baseline) |  |  |  |  |  |  |  |  | |  | |  | |  | |  | |  |
| N | 145 | 28 | 113 | 22 | 23 | 6 | 24 | 4 | | 21 | | 2 | | 3 | | 1 | |  |
| GMC (95% CI) | <LLOQ (<LLOQ–40) | <LLOQ (<LLOQ–38) | <LLOQ (<LLOQ–45) | 38 (<LLOQ–63) | <LLOQ (<LLOQ–46) | <LLOQ (<LLOQ–  <LLOQ) | 41 (<LLOQ–77) | 41 (<LLOQ–535) | | 57 (<LLOQ–120) | | <LLOQ (<LLOQ–  <LLOQ) | | <LLOQ (<LLOQ–  <LLOQ) | | <LLOQ | |  |
|  |  |  |  |  |  |  |  |  | |  | |  | |  | |  | |  |
| Day 29 (28 days post-dose 1) |  |  |  |  |  |  |  |  | |  | |  | |  | |  | |  |
| N | 149 | 28 | 0 | 0 | 0 | 0 | 24 | 4 | | 0 | | 0 | | 0 | | 0 | |  |
| GMC (95% CI) | 327 (276–387) | <LLOQ (<LLOQ–40) | - | - | - | - | 366 (214–626) | 40 (<LLOQ–526) | | - | | - | | - | | - | |  |
| Responder *n*/N* (%) | 113/145 (77.9) | 0/28 | - | - | - | - | 17/24 (70.8) | 0/4 | | - | | - | | - | | - | |  |
| 95% CI (%) | (70.3–84.4) | (0–12.3) | - | - | - | - | (48.9–87.4) | (0–60.2) | | - | | - | | - | | - | |  |
|  |  |  |  |  |  |  |  |  | |  | |  | |  | |  | |  |
| Day 50 (21 days post-dose 2) |  |  |  |  |  |  |  |  | |  | |  | |  | |  | |  |
| N | 147 | 26 | 0 | 0 | 0 | 0 | 24 | 4 | | 0 | | 0 | | 0 | | 0 | |  |
| GMC (95% CI) | 3339 (2849–3914) | <LLOQ (<LLOQ–  <LLOQ) | - | - | - | - | 1900 (1184–3050) | 40 (<LLOQ–487) | | - | | - | | - | | - | |  |
| Responder *n*/N* (%) | 141/143 (98.6) | 0/26 | - | - | - | - | 23/24 (95.8) | 0/4 | | - | | - | | - | | - | |  |
| 95% CI (%) | (95–99.8) | (0–13.2) | - | - | - | - | (78.9–99.9) | (0–60.2) | | - | | - | | - | | - | |  |
|  |  |  |  |  |  |  |  |  | |  | |  | |  | |  | |  |
| Day 57 (56 days post-dose 1) |  |  |  |  |  |  |  |  | |  | |  | |  | |  | |  |
| N | 0 | 0 | 114 | 22 | 0 | 0 | 0 | 0 | | 22 | | 2 | | 0 | | 0 | |  |
| GMC (95% CI) | - | - | 365 (308–433) | 40 (<LLOQ–67) | - | - | - | - | | 337 (211–538) | | <LLOQ (<LLOQ–  <LLOQ) | | - | | - | |  |
| Responder *n*/N* (%) | - | - | 92/112 (82.1) | 1/22 (4.5) | - | - | - | - | | 15/21 (71.4) | | 0/2 | | - | | - | |  |
| 95% CI (%) | - | - | (73.8–88.7) | (0.1–22.8) | - | - | - | - | | (47.8–88.7) | | (0–84.2) | | - | | - | |  |
|  |  |  |  |  |  |  |  |  | |  | |  | |  | |  | |  |
| Day 78 (21 days post-dose 2) |  |  |  |  |  |  |  |  | |  | |  | |  | |  | |  |
| N | 0 | 0 | 115 | 22 | 0 | 0 | 0 | 0 | | 21 | | 2 | | 0 | | 0 | |  |
| GMC (95% CI) | - | - | 8113 (6875–9573) | <LLOQ (<LLOQ–58) | - | - | - | - | | 4956 (3564–6891) | | <LLOQ (<LLOQ–  <LLOQ) | | - | | - | |  |
| Responder *n*/N* (%) | - | - | 113/113 (100) | 1/22 (4.5) | - | - | - | - | | 19/20 (95) | | 0/2 | | - | | - | |  |
| 95% CI (%) | - | - | (96.8–100) | (0.1–22.8) | - | - | - | - | | (75.1–99.9) | | (0–84.2) | | - | | - | |  |
|  |  |  |  |  |  |  |  |  | |  | |  | |  | |  | |  |
| Day 85 (84 days post-dose 1) |  |  |  |  |  |  |  |  | |  | |  | |  | |  | |  |
| N | 0 | 0 | 0 | 0 | 24 | 6 | 0 | 0 | | 0 | | 0 | | 3 | | 1 | |  |
| GMC (95% CI) | - | - | - | - | 247 (180–340) | <LLOQ (<LLOQ–  <LLOQ) | - | - | | - | | - | | 204 (50–828) | | <LLOQ | |  |
| Responder *n*/N* (%) | - | - | - | - | 18/23 (78.3) | 0/6 | - | - | | - | | - | | 3/3 (100) | | 0/1 | |  |
| 95% CI (%) | - | - | - | - | (56.3–92.5) | (0–45.9) | - | - | | - | | - | | (29.2–100) | | (0–97.5) | |  |
|  |  |  |  |  |  |  |  |  | |  | |  | |  | |  | |  |
| Day 106 (21 days post-dose 2) |  |  |  |  |  |  |  |  | |  | |  | |  | |  | |  |
| N | 0 | 0 | 0 | 0 | 24 | 6 | 0 | 0 | | 0 | | 0 | | 3 | | 1 | |  |
| GMC (95% CI) | - | - | - | - | 8086 (5546–11790) | <LLOQ (<LLOQ–<LLOQ) | - | - | | - | | - | | 3221 (1057–9815) | | <LLOQ | |  |
| Responder *n*/N* (%) | - | - | - | - | 23/23 (100) | 0/6 | - | - | | - | | - | | 3/3 (100) | | 0/1 | |  |
| 95% CI (%) | - | - | - | - | (85.2–100) | (0–45.9) | - | - | | - | | - | | (29.2–100) | | (0–97.5) | |  |
|  |  |  |  |  |  |  |  |  | |  | |  | |  | |  | |  |
| Day 365 (364 days post-dose 1) |  |  |  |  |  |  |  |  | |  | |  | |  | |  | |  |
| N | 143 | 27 | 112 | 22 | 24 | 6 | 24 | 4 | | 21 | | 2 | | 3 | | 1 | |  |
| GMC (95% CI) | 320 (275–373) | <LLOQ (<LLOQ–  <LLOQ) | 349 (291–417) | <LLOQ (<LLOQ–49) | 369 (225–606) | <LLOQ (<LLOQ–  <LLOQ) | 272 (171–432) | 38 (<LLOQ–400) | | 307 (211–446) | | <LLOQ (<LLOQ–  <LLOQ) | | 317 (178–567) | | <LLOQ | |  |
| Responder *n*/N* (%) | 113/139 (81.3) | 0/27 | 87/110 (79.1) | 0/22 | 20/23 (87) | 0/6 | 17/24 (70.8) | 0/4 | | 14/20 (70) | | 0/2 | | 3/3 (100) | | 0/1 | |  |
| 95% CI (%) | (73.8–87.4) | (0–12.8) | (70.3–86.3) | (0–15.4) | (66.4–97.2) | (0–45.9) | (48.9–87.4) | (0–60.2) | | (45.7–88.1) | | (0–84.2) | | (29.2–100) | | (0–97.5) | |  |
|  |  |  |  |  |  |  |  |  | |  | |  | |  | |  | |  |
| Day 365 (Pre-booster) |  |  |  |  |  |  |  |  | |  | |  | |  | |  | |  |
| N | 26 | 7 | 28 | 8 | 0 | 0 | 6 | 1 | | 11 | | 0 | | 0 | | 0 | |  |
| GMC (95% CI) | 302 (206–443) | <LLOQ (<LLOQ–  <LLOQ) | 352 (239–519) | <LLOQ (<LLOQ–62) | - | - | 298 (111–804) | <LLOQ | | 406 (269–612) | | - | | - | | - | |  |
|  |  |  |  |  |  |  |  |  | |  | |  | |  | |  | |  |
| Day 369 (4 days post-booster) |  |  |  |  |  |  |  |  | |  | |  | |  | |  | |  |
| N | 27 | 7 | 28 | 9 | 0 | 0 | 6 | 1 | | 11 | | 0 | | 0 | | 0 | |  |
| GMC (95% CI) | 376 (250–565) | <LLOQ (<LLOQ–37) | 517 (341–785) | <LLOQ (<LLOQ–52) | - | - | 439 (134–1442) | <LLOQ | | 647 (406–1030) | | - | | - | | - | |  |
| Responder *n*/N* (%) | 19/25 (76) | 0/7 | 21/27 (77.8) | 0/9 | - | - | 5/6 (83.3) | 0/1 | | 6/10 (60) | | - | | - | | - | |  |
| 95% CI (%) | (54.9–90.6) | (0–41) | (57.7–91.4) | (0–33.6) | - | - | (35.9–99.6) | (0–97.5) | | (26.2–87.8) | | - | | - | | - | |  |
|  |  |  |  |  |  |  |  |  | |  | |  | |  | |  | |  |
| Day 372 (7 days post-booster) |  |  |  |  |  |  |  |  | |  | |  | |  | |  | |  |
| N | 27 | 7 | 28 | 9 | 0 | 0 | 6 | 1 | | 11 | | 0 | | 0 | | 0 | |  |
| GMC (95% CI) | 16763 (12157–23116) | <LLOQ (<LLOQ–  <LLOQ) | 22259 (15552–31857) | <LLOQ (<LLOQ–50) | - | - | 16088 (7247–35713) | <LLOQ | | 16385 (10441–25712) | | - | | - | | - | |  |
| Responder *n*/N* (%) | 25/25 (100) | 0/7 | 27/27 (100) | 0/9 | - | - | 6/6 (100) | 0/1 | | 10/10 (100) | | - | | - | | - | |  |
| 95% CI (%) | (86.3–100) | (0–41) | (87.2–100) | (0–33.6) | - | - | (54.1–100) | (0–97.5) | | (69.2–100) | | - | | - | | - | |  |
|  |  |  |  |  |  |  |  |  | |  | |  | |  | |  | |  |
| Day 386 (21 days post-booster) |  |  |  |  |  |  |  |  | |  | |  | |  | |  | |  |
| N | 27 | 7 | 28 | 9 | 0 | 0 | 6 | 1 | | 11 | | 0 | | 0 | | 0 | |  |
| GMC (95% CI) | 29450 (19549–44366) | <LLOQ (<LLOQ–  <LLOQ) | 40341 (29721–54756) | <LLOQ (<LLOQ–53) | - | - | 28716 (11703–70458) | <LLOQ | | 45151 (24794–82221) | | - | | - | | - | |  |
| Responder *n*/N* (%) | 25/25 (100) | 0/7 | 27/27 (100) | 1/9 (11.1) | - | - | 6/6 (100) | 0/1 | | 10/10 (100) | | - | | - | | - | |  |
| 95% CI (%) | (86.3–100) | (0–41) | (87.2–100) | (0.3–48.2) | - | - | (54.1–100) | (0–97.5) | | (69.2–100) | | - | | - | | - | |  |
|  |  |  |  |  |  |  |  |  | |  | |  | |  | |  | |  |
| Day 729 (364 days post-booster) |  |  |  |  |  |  |  |  | |  | |  | |  | |  | |  |
| N | 27 | 7 | 26 | 9 | 0 | 0 | 5 | 1 | | 11 | | 0 | | 0 | | 0 | |  |
| GMC (95% CI) | 4671 (2865–7615) | <LLOQ (<LLOQ–  <LLOQ) | 4580 (2855–7346) | <LLOQ (<LLOQ–37) | - | - | 3857 (750–19832) | <LLOQ | | 3950 (1747–8930) | | - | | - | | - | |  |
| Responder *n*/N* (%) | 25/25 (100) | 0/7 | 25/25 (100) | 0/9 | - | - | 5/5 (100) | 0/1 | | 9/10 (90) | | - | | - | | - | |  |
| 95% CI (%) | (86.3–100) | (0–41) | (86.3–100) | (0–33.6) | - | - | (47.8–100) | (0–97.5) | | (55.5–99.7) | | - | | - | | - | |  |

Vaccines: Ad26 = Ad26.ZEBOV at a dose of 5x10^10^ vp; MVA = MVA-BN-Filo at a dose of 1x10^8^ Inf.U.

CI, confidence interval; EBOV GP, Ebola virus glycoprotein; GMC, geometric mean concentration; Inf.U, Infectious Units; LLOQ, lower limit of quantification; *n,* number of responders; N, number of participants with data at that timepoint; N*, number of participants with data at baseline and at that timepoint; vp, viral particles.

A participant was a responder at a considered timepoint if the sample interpretation was negative at baseline and positive post-baseline and the post-baseline value was greater than 2.5x LLOQ, or sample interpretation was positive both at baseline and post-baseline and there was a greater than 2.5-fold increase from baseline. The GMC and corresponding CI are shown on the reported scale (ELISA Units/mL); the Exact Clopper-Pearson Confidence Interval is shown for the corresponding responder rate; who did not receive dose 2 are excluded from the sensitivity analysis.

| **Table F. EBOV-GP-specific binding antibodies in healthy adult participants in the active vaccine groups – per-protocol analysis set.** | | | | | | | | | |
| --- | --- | --- | --- | --- | --- | --- | --- | --- | --- |
|  | | **Two-dose primary regimens** | | | **One-year persistence and booster** | | | **Two-year persistence (one year after Ad26.ZEBOV booster)** |  |
|  | | **Baseline** | **Before**  **dose 2**  **(MVA-BN-Filo)** | **21 days after dose 2**  **(MVA-BN-Filo)** | **One-year persistence** | **After Ad26.ZEBOV booster** | |  |  |
|  |  |  |  |  |  | **7 days** | **21 days** |  |  |
| **Group 1: 28-day interval** | | | | | | | | | |
| **Day** | | **1** | **29** | **50** | **365** | **372** | **386** | **729** |  |
| N | | 169 | 173 | 171 | 167 | 33 | 33 | 32 |  |
| GMC  (95% CI) | | <LLOQ  (<LLOQ–41) | 332  (282–390) | 3085  (2648–3594) | 313  (271–361) | 16 639  (12 567–22 030) | 29 315  (20 614–41 689) | 4534  (2911–7060) |  |
| Responders | *n*/N* (%) | - | 130/169 (77) | 164/167 (98) | 130/163 (80) | 31/31 (100) | 31/31 (100) | 30/30 (100) |  |
| **Group 2: 56-day interval** | | | | | | | | | |
| **Day** | | **1** | **57** | **78** | **365** | **372** | **386** | **729** |  |
| N | | 134 | 136 | 136 | 133 | 39 | 39 | 37 |  |
| GMC  (95% CI) | | 39  (<LLOQ–48) | 361  (307–423) | 7518  (6468–8740) | 342  (291–401) | 20416  (15 432–27 009) | 41 643  (32 045–54 116) | 4383  (2969–647) |  |
| Responders | *n*/N* (%) | - | 107/133 (80) | 132/133 (99) | 101/130 (78) | 37/37 (100) | 37/37 (100) | 34/35 (97) |  |
| **Group 3: 84-day interval** | | | | | | | | | |
| **Day** | | **1** | **85** | **106** | **365** | **372** | **386** | **729** |  |
| N | | 26 | 27 | 27 | 27 |  |  |  |  |
| GMC  (95% CI) | | 26  (<LLOQ–41) | 242  (181–323) | 7300  (5116–10 417) | 363  (234–562) | NA | NA | NA |  |
| Responders | *n*/N* (%) | - | 21/26 (81) | 26/26 (100) | 23/26 (88) |  |  |  |  |
| One participant in the 56-day placebo group showed antibody concentrations above LLOQ at Days 57, 78, and 386. Nevertheless, all GMCs were <LLOQ in all placebo groups, and therefore GMCs are not shown.  CI, confidence interval; EBOV GP, Ebola virus glycoprotein; GMC, geometric mean concentration; <LLOQ, below the lower limit of quantification; NA, not applicable; N, number of participants with data at that timepoint; N*, number of participants with data at baseline and at that time point. | | | | | | | | | |

|  | **Table G. Comparison of EBOV-GP-specific binding antibodies in healthy adult and HIV-infected adults in the active vaccine groups – per-protocol analysis set** | | | | | |
| --- | --- | --- | --- | --- | --- | --- |
|  | | | Comparison | Difference (95% CI) | GMC Ratio (95% CI) | P-value |
| Healthy adults | | 21 days post-dose 2 | Group1 vs. Group 2 vs. Group 3* | - | - | <0.001 |
|  |  |  | Group 1 vs. Group 2 | -0.39 (-0.50 – -0.27) | 0.4 (0.3-0.5) | <0.001 |
|  |  |  | Group 1 vs. Group 3 | -0.37 (-0.58 – -0.17) | 0.4 (0.3-0.7) | <0.001 |
|  |  |  | Group 2 vs. Group 3 | 0.01 (-0.19 – 0.22) | 1.0 (0.6-1.6) | 1.00 |
|  |  | 1-year post-dose 1 | Group1 vs. Group 2 vs. Group 3* | - | - | 0.64 |
|  |  |  | Group 1 vs. Group 2 | -0.04 (-0.16 – 0.08) | 0.9 (0.7-1.2) | 1.00 |
|  |  |  | Group 1 vs. Group 3 | -0.06 (-0.31 – 0.18) | 0.9 (0.5-1.5) | 1.00 |
|  |  |  | Group 2 vs. Group 3 | -0.03 (-0.27 – 0.22) | 0.9 (0.5-1.7) | 1.00 |
|  | |  |  |  |  |  |
| HIV-infected adults | | 21 days post-dose 2 | Group 1 vs. Group 2 | -0.10 (-0.26 – 0.06) | 0.8 (0.6-1.1) | 0.22 |
|  |  | 1-year post-dose 1 | Group 1 vs. Group 2 | 0.13 (-0.03 – 0.30) | 1.4 (0.9-2.0) | 0.12 |
| *Global test (F-test) of the null hypothesis that the Group means (log_10_-scale) are all equal versus the alternative hypothesis that at least one of the Group means differ. Bonferroni adjustment for the *p*-values and confidence intervals for the differences of pairwise comparisons for healthy adults was implemented at 21 days post-dose 2 and again at 1-year post-dose 1.  CI, confidence interval; EBOV GP, Ebola virus glycoprotein.  The computations are based on the log_10_-transformed data and assumed unequal variances in the comparison groups, the GMC ratio and its 95% CIs are obtained by back-transforming the estimated difference (on log_10_-transformed data) and its 95% CI. Group 1: 28-day interval. Group 2: 56-day interval. Group 3: 84-day interval. | | | | | | |
|  |  |  |  |  |  |  |

| **Table H. EBOV-GP-specific binding antibodies in HIV-infected adults in the active vaccine groups – per-protocol analysis set.** | | | | | | |
| --- | --- | --- | --- | --- | --- | --- |
|  | | **Two-dose primary regimens** | | |  | **One-year persistence** |
|  | | **Baseline** | **Before dose 2**  **(MVA-BN-Filo)** | **21 days after dose 2**  **(MVA-BN-Filo)** |  |  |
| **Group 1: 28-day interval** | | | | | | |
| **Day** | | **1** | **29** | **50** |  | **365** |
| N | | 58 | 58 | 58 |  | 56 |
| GMC  (95% CI) | | <LLOQ  (<LLOQ–38) | 368  (272–497) | 4207  (3233–5474) |  | 459  (352–600) |
| Responders | *n*/N*  (%) | NA | 47/58  (81) | 58/58  (100) |  | 48/56  (86) |
| **Group 2: 56-day interval** | | | | | | |
| **Day** | | **1** | **57** | **78** |  | **365** |
| N | | 58 | 59 | 59 |  | 59 |
| GMC  (95% CI) | | 39  (<LLOQ–<LLOQ) | 291  (233–364) | 5283  (4094–6817) |  | 338  (253–450) |
| Responders | *n*/N*  (%) | NA | 51/58  (88) | 58/58  (100) |  | 51/58  (88) |
| One participant in the 28-day placebo group showed an antibody concentration above LLOQ at Day 29. Nevertheless, all GMCs were <LLOQ in all placebo groups, and therefore GMCs are not shown.  CI, confidence interval; EBOV GP, Ebola virus glycoprotein; GMC, geometric mean concentration; HIV, human immunodeficiency virus; <LLOQ, below the lower limit of quantification; *n*, number of responders; N, number of participants with data at that timepoint; N*, number of participants with data at baseline and at that time point; NA, not applicable. | | | | | | |

| **Table I. Comparison of EBOV-GP-specific binding antibodies in healthy adult versus HIV-infected adult participants in the active vaccine groups – per-protocol analysis set** | | | | | |
| --- | --- | --- | --- | --- | --- |
|  |  | **Comparison** | **Difference (95% CI)** | **GMC Ratio (95% CI)** | **P-value** |
| **Group 1: 28-day interval** | 21 days post-dose 2 | Healthy adults  vs.  HIV-infected adults | -0.13 (-0.27– - 0.003) | 0.7 (0.5-0.99) | 0.04 |
|  | 1-year post-dose 1 | Healthy adults  vs.  HIV-infected adults | -0.17 (-0.30– -0.04) | 0.7 (0.5–0.92) | 0.01 |
| **Group 2: 56-day interval** | 21 days post-dose 2 | Healthy adults  vs.  HIV-infected adults | 0.15 (0.03–0.28) | 1.4 (1.1-1.9) | 0.02 |
|  | 1-year post-dose 1 | Healthy adults  vs.  HIV-infected adults | 0.01 (-0.14– 0.15) | 1.0 (0.7–1.4) | 0.94 |
| The computations are based on the log_10_-transformed data and assumed unequal variances in the comparison groups, the GMC ratio and its 95% CIs are obtained by back-transforming the estimated difference (on log_10_-transformed data) and its 95% CI.  CI, confidence interval; EBOV GP, Ebola virus glycoprotein. | | | | | |

| **Table J.** **EBOV GP-specific binding antibodies in healthy adults receiving the second dose of active vaccine outside the protocol-defined window** | | | |  |
| --- | --- | --- | --- | --- |
| **Vaccination Schedule** | **Baseline** | **Pre-dose 2 a** | **21 Days Post-dose 2** |  |
| **140-day interval** |  |  |  |  |
| Ad26, MVA |  |  |  |  |
| N | 19 | 19 | 17 |  |
| GMC (95% CI) | <LLOQ (<LLOQ–37) | 266 (198–358) | 15 555 (10 907–22 184) |  |
| Responders *n*/N* (%) | - | 16/19 (84.2) | 17/17 (100.0) |  |
| (95% CI [%]) | - | (60.4–96.6) | (80.5–100.0) |  |
| **196-day interval** |  |  |  |  |
| Ad26, MVA |  |  |  |  |
| N | 49 | 52 | 52 |  |
| GMC (95% CI) | <LLOQ (<LLOQ–43) | 197 (159–245) | 14 995 (11 855–18 965) |  |
| Responders *n*/N* (%) | - | 33/49 (67.3) | 49/49 (100.0) |  |
| (95% CI [%]) | - | (52.5–80.1) | (92.7–100.0) |  |
| **252-day interval** |  |  |  |  |
| Ad26, MVA |  |  |  |  |
| N | 75 | 76 | 77 |  |
| GMC (95% CI) | 40 (<LLOQ–52) | 239 (195–293) | 16 149 (13 882–18 786) |  |
| Responders *n*/N* (%) | - | 53/74 (71.6) | 75/75 (100.0) |  |
| (95% CI [%]) | - | (59.9–81.5) | (95.2–100.0) |  |
| **≥280-day interval** |  |  |  |  |
| Ad26, MVA |  |  |  |  |
| N | 21 | 21 | 21 |  |
| GMC (95% CI) | 39 (<LLOQ–72) | 212 (121–372) | 23 897 (16 703–34 188) |  |
| Responders *n*/N* (%) | - | 11/21 (52.4) | 21/21 (100.0) |  |
| (95% CI [%]) | - | (29.8–74.3) | (83.9–100.0) |  |
|  | | | | |

Definition of vaccination schedules outside the protocol defined window: 140-day interval, window ranging from 99 to 168 days between dose 1 (i.e. vaccination at Day 1) and dose 2; 196-day interval, window ranging from 169 to 224 days between dose 1 and dose 2; 252-day interval, window ranging from 225 to 279 days between dose 1 and dose 2; ≥280-day interval, window of ≥280 days between dose 1 and dose 2.

A participant was a responder at a considered timepoint if the sample interpretation was negative at baseline and positive post-baseline and the post-baseline value was greater than 2.5x LLOQ, or sample interpretation was positive both at baseline and post-baseline and there was a greater than 2.5-fold increase from baseline.

The GMC and corresponding CI are shown on the reported scale (ELISA units/mL); the Exact Clopper-Pearson CI is shown for the corresponding responder rate; ^a^ pre-dose 2 datapoint closest to target day; participants who did not receive dose 2 are excluded from the sensitivity analysis.

At pre-dose 2, one participant in the 196-day interval placebo group showed an antibody concentration above LLOQ; within this placebo group GMCs were above the LLOQ at baseline (43 ELISA units/mL), pre-dose 2 (57 ELISA units/mL), and 21 days post-dose 2 (39 ELISA units/mL).

At pre-dose 2 and 21 days post-dose 2, one participant in the 252-day interval placebo group showed an antibody concentration above LLOQ; within this placebo group GMCs were above the LLOQ at baseline (60 ELISA units/mL), pre-dose 2 (49 ELISA units/mL), and 21 days post-dose 2 (57 ELISA units/mL). All GMCs were <LLOQ in the remaining placebo groups.

Vaccines: Ad26 = Ad26.ZEBOV at a dose of 5x10^10^ vp; MVA = MVA-BN-Filo at a dose of 1x10^8^ Inf.U.

CI, confidence interval; EBOV GP, Ebola virus glycoprotein; GMC, geometric mean concentration; Inf.U; Infectious Units; LLOQ, lower limit of quantification; *n,* number of responders; N, number of participants with data at that timepoint; N*, number of participants with data at baseline and at  that timepoint; vp, viral particles.

| **Table K. GMT of EBOV GP-specific pseudovirion neutralising antibodies titres (IC_50_) and responder rates in healthy adult active vaccine groups 1 and 2 – per-protocol analysis set.** | | | | | |
| --- | --- | --- | --- | --- | --- |
|  | | **Two-dose primary regimens** | |  | **One-year persistence** |
|  | | **Baseline** | **21 days after**  **dose 2**  **(MVA-BN-Filo)** |  |  |
| **Group 1: 28-day interval** | | | | | |
| **Day** | | **1** | **50** |  | **365** |
| N | | 39 | 39 |  | 39 |
| GMT  (95% CI) | | <LLOQ  (<LLOQ–<LLOQ) | 982  (714–1350) |  | 123  (<LLOQ–165) |
| Responders | *n*/N*  (%) | - | 36/39  (92) |  | 8/39  (21) |
| **Group 2: 56-day interval** | | | | | |
| **Day** | | **1** | **78** |  | **365** |
| N | | 38 | 37 |  | 37 |
| GMT  (95% CI) | | <LLOQ  (<LLOQ–<LLOQ) | 4100  (2927–5745) |  | 153  (<LLOQ–210) |
| Responders | *n*/N*  (%) | - | 36/37  (97) |  | 9/37  (24) |
| In the placebo groups all titers were <LLOQ with zero responder rates.  CI, confidence interval; EBOV GP, Ebola virus glycoprotein; GMT, geometric mean titre; <LLOQ, below the lower limit of quantification; *n,* number of responders; N, number of participants with data at that timepoint; N*, number of participants with data at baseline and at that time point. | | | | | |

| **Table L. Ad26 neutralising antibodies (Ad26 VNA, IC_90_ titer) in healthy and HIV-infected adults – per protocol analysis set** | | | | | | | | |
| --- | --- | --- | --- | --- | --- | --- | --- | --- |
|  | **Healthy adults** | | | | **HIV-infected adults** | | | |
|  | 28-day interval | | 56-day interval | | 28-day interval | | 56-day interval | |
|  | **Ad26, MVA** | **Placebo, Placebo** | **Ad26, MVA** | **Placebo, Placebo** | **Ad26, MVA** | **Placebo, Placebo** | **Ad26, MVA** | **Placebo, Placebo** |
| Day 1 (Baseline) |  |  |  |  |  |  |  |  |
| N | 39 | 9 | 38 | 12 | 58 | 11 | 59 | 12 |
| GMT (95% CI) | 90 (52–157) | 63 (25–162) | 129 (84–196) | 142 (67–299) | 83 (54–129) | 70 (31–158) | 76 (53–109) | 62 (25–154) |
| Positive Sample, *n* (%) | 32 (82.1) | 8 (88.9) | 37 (97.4) | 12 (100.0) | 45 (77.6) | 10 (90.9) | 51 (86.4) | 11 (91.7) |
| (95% CI [%]) | (66.5–92.5) | (51.8–99.7) | (86.2–99.9) | (73.5–100.0) | (64.7–87.5) | (58.7–99.8) | (75.0–94.0) | (61.5–99.8) |
| Day 365 (pre-booster) |  |  |  |  |  |  |  |  |
| N | 21 | 4 | 30 | 6 | - | - | - | - |
| GMT (95% CI) | 1208 (601–2427) | 85 (50–143) | 1138 (723–1792) | 69 (<LLOQ–287) |  |  |  |  |
| Positive Sample, *n* (%) | 21 (100.0) | 4 (100.0) | 30 (100.0) | 5 (83.3) |  |  |  |  |
| (95% CI [%]) | (83.9–100.0) | (39.8–100.0) | (88.4–100.0) | (35.9–99.6) |  |  |  |  |

The geometric mean titer and corresponding confidence interval are shown on the reported scale (IC_90_ titer). Exact Clopper-Pearson confidence interval is shown for the corresponding sample interpretation rate.

Vaccines: Ad26 = Ad26.ZEBOV at a dose of 5x10^10^ vp; MVA = MVA-BN-Filo at a dose of 1x10^8^ Inf.U.

Ad26 VNA, Ad26-specific virus neutralisation assay; CI, confidence interval; GMT, geometric mean titer; HIV, human immunodeficiency virus; Inf.U, Infectious Units; N, number of participants with data at that timepoint; vp, viral particles.

| **Table M. EBOV GP-specific IFN- γ producing T cell responses (IFN- γ ELISpot) in healthy and HIV-infected adults – per protocol analysis set** | | | | | | | |
| --- | --- | --- | --- | --- | --- | --- | --- |
|  |  | **Healthy adults** | | | | **HIV-infected adults** | |
|  |  | 28-day interval | | 56-day interval | | 56-day interval | |
| **Study injections** | Dose 1 | **Ad26** | **Placebo** | **Ad26** | **Placebo** | **Ad26** | **Placebo** |
|  | Dose 2 | **MVA** | **Placebo** | **MVA** | **Placebo** | **MVA** | **Placebo** |
| **IFNγ (ELISpot)**  N  Median reportable value (SFU/10^6^ PBMC)  (Q1; Q3)  Responder rate (%) | **Day 1** | 34  <50  (<50–<50) | 9  <50  (<50–<50) | 31  <50  (<50–<50) | 7  <50  (<50–203) | 12  <50  (<50–<50) | 2  <50  (<50–<50) |
|  | **Day 50*** | 35  73  (<50–148)  27 | 9  <50  (<50–<50)  0 | -  -  -  - | -  -  -  - | -  -  -  - | -  -  -  - |
|  | **Day 78*** | -  -  -  - | -  -  -  - | 30  61  (<50–105)  27 | 7  <50  (<50–385)  0 | 13  <50  (<50–105]  17 | 2  <50  (<50–<50)  0 |
|  | **Day 365** | 36  <50  (<50–113]  18 | 8  <50  (<50–<50)  0 | 30  <50  (<50–68)  7 | 7  <50  (<50–237)  0 | 13  <50  (<50–58)  17 | 2  <50  (<50–<50)  0 |

*21 days post-dose 2 timepoint.

Vaccines: Ad26 = Ad26.ZEBOV at a dose of 5x10^10^ vp; MVA = MVA-BN-Filo at a dose of 1x10^8^ Inf.U.

EBOV GP, Ebola virus glycoprotein; HIV, human immunodeficiency virus; IFN-γ, Interferon gamma; SFU/10^6^ PBMC, spot-forming units per million peripheral blood mononuclear cells; N, number of participants with data at that timepoint.
