# Supplementary material for: Safety and immunogenicity of 2-dose heterologous Ad26.ZEBOV, MVA-BN-Filo Ebola vaccination in healthy and HIV-infected adults: A randomised, placebo-controlled Phase II clinical trial in Africa
Source: PLoS Med. 2021 Oct 29;18(10):e1003813. doi: 10.1371/journal.pmed.1003813 (PMC8555783; doi:10.1371/journal.pmed.1003813)
Supplement: S1 Fig — (A) 21 days post-dose 2. (B) 364 days post-dose 2. EBOV GP, Ebola virus glycoprotein; LLOQ, lower limit of quantification; ULOQ, upper limit of quantification. (DOCX) [file pmed.1003813.s009.docx]

**S1 Fig.** **Correlations between EBOV GP-specific binding and neutralising antibodies in participants assigned to an active regimen:**

**A) 21 days post-dose 2**

**B) 364 days post-dose 2**


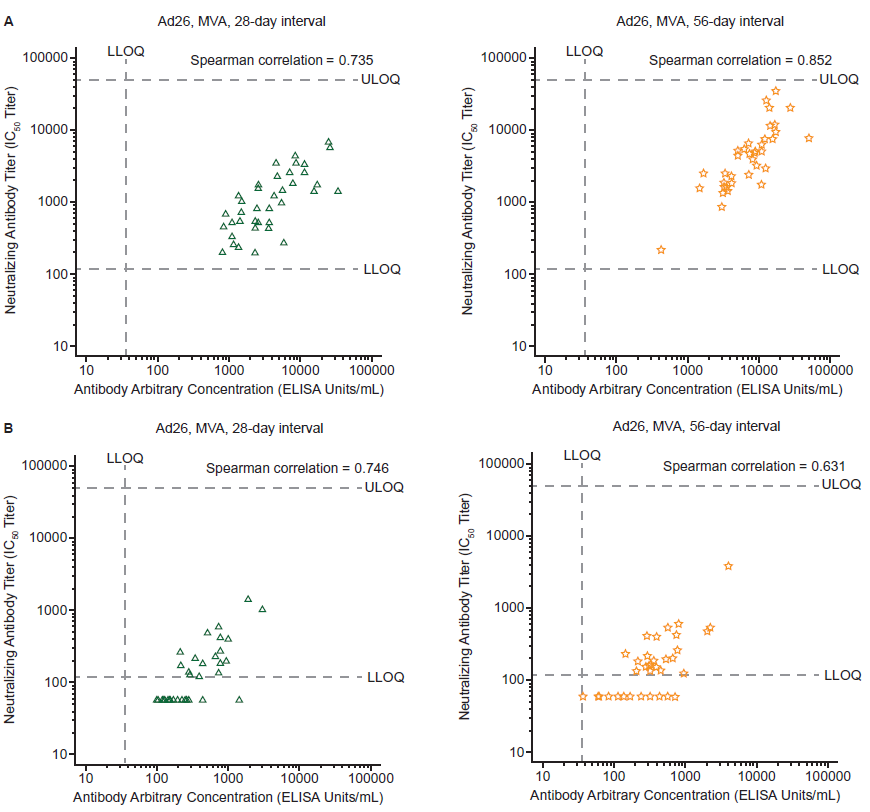


Vaccines: Ad26 = Ad26.ZEBOV at a dose of 5x10^10^ vp; MVA = MVA-BN-Filo at a dose of 1x10^8^ Inf.U.

EBOV GP = Ebola virus glycoprotein; LLOQ = lower limit of quantification; ULOQ = upper limit of quantification.
