# Supplementary material for: Safety and immunogenicity of 2-dose heterologous Ad26.ZEBOV, MVA-BN-Filo Ebola vaccination in healthy and HIV-infected adults: A randomised, placebo-controlled Phase II clinical trial in Africa
Source: PLoS Med. 2021 Oct 29;18(10):e1003813. doi: 10.1371/journal.pmed.1003813 (PMC8555783; doi:10.1371/journal.pmed.1003813)
Supplement: S2 Fig — (A) Anti-EBOV GP IgG ELISA at 21 days post-dose 2 by Ad26 neutralisation assay at baseline. (B) EBOV GP neutralisation assay at 21 days post-dose 2 by Ad26 neutralisation assay at baseline. Ad26 VNA, Ad26-specific virus neutralisation assay; EBOV GP, Ebola virus glycoprotein; ELISA, enzyme-linked immunosorbent assay; IgG, immunoglobulin G; LLOQ, lower limit of quantification; ULOQ, upper limit of quantification. (DOCX) [file pmed.1003813.s010.docx]

**S2 Fig. Correlations between Ad26-specific neutralising antibody titres at baseline and EBOV GP-specific binding and neutralising antibodies 21 days post-dose 2 in healthy adults.**

**A: Anti EBOV GP IgG ELISA at 21 days post-dose 2 by Ad26 neutralisation assay at baseline.**

**B: EBOV GP neutralisation assay** **at 21 days post-dose 2 by Ad26 neutralisation assay at baseline.**


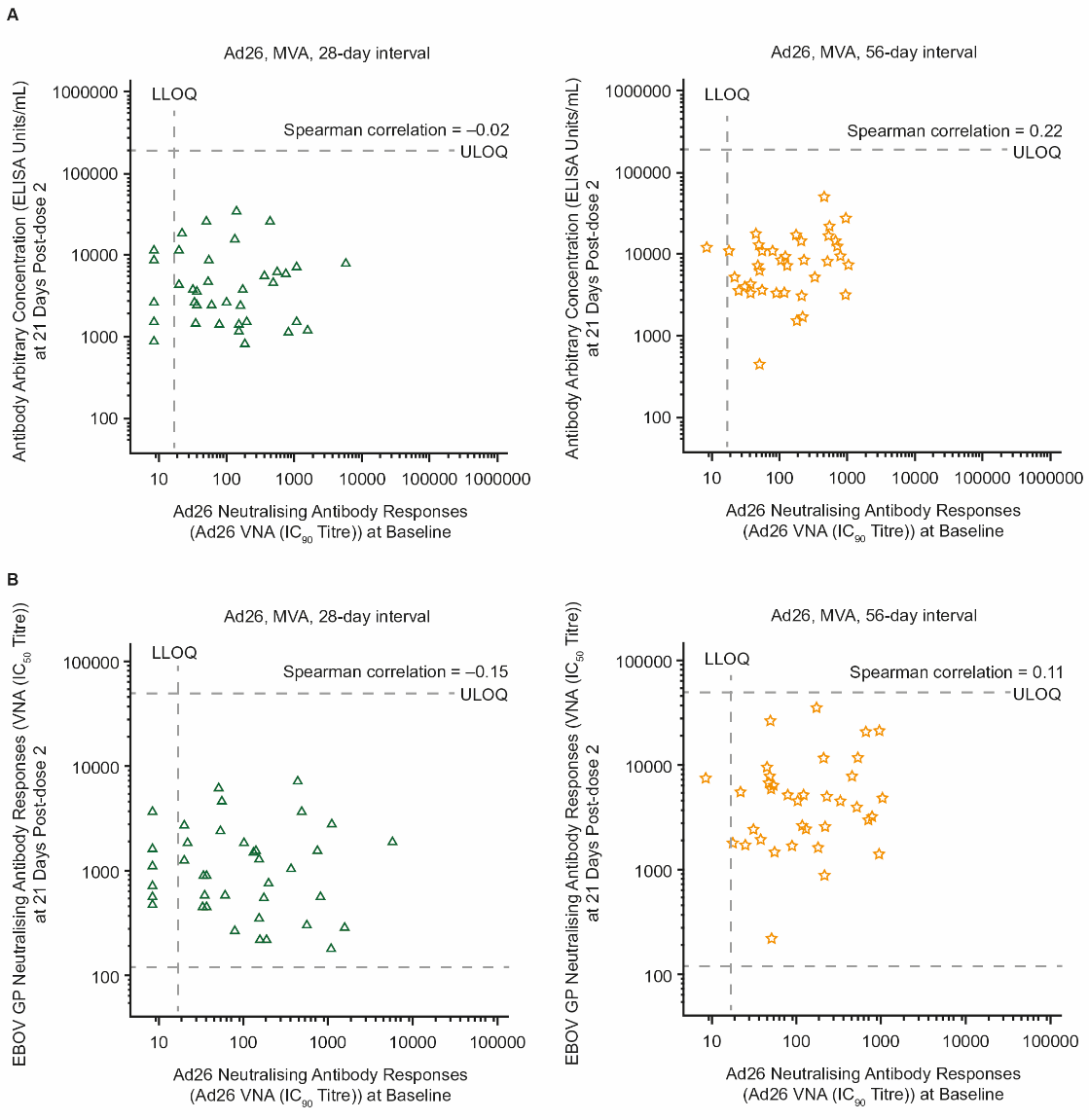


Placebo recipients are excluded from this display.

Vaccines: Ad26 = Ad26.ZEBOV at a dose of 5x10^10^ vp; MVA = MVA-BN-Filo at a dose of 1x10^8^ Inf.U.

Ad26 VNA = Ad26-specific virus neutralisation assay; EBOV GP = Ebola virus glycoprotein; ELISA = enzyme-linked immunosorbent assay; IgG = immunoglobulin G; LLOQ = lower limit of quantification; ULOQ = upper limit of quantification
