# Supplementary material for: Safety and immunogenicity of 2-dose heterologous Ad26.ZEBOV, MVA-BN-Filo Ebola vaccination in healthy and HIV-infected adults: A randomised, placebo-controlled Phase II clinical trial in Africa
Source: PLoS Med. 2021 Oct 29;18(10):e1003813. doi: 10.1371/journal.pmed.1003813 (PMC8555783; doi:10.1371/journal.pmed.1003813)
Supplement: S4 Fig — EBOV GP–specific CD4+ (A) and CD8+ (B) T cell cytokine responses in healthy adult participants. EBOV GP, Ebola virus glycoprotein; n, number of participants with data at that time point; NA, not applicable. (DOCX) [file pmed.1003813.s012.docx]

**S4 Fig. EBOV GP-specific CD4+ (A) and CD8+ (B) T cell cytokine responses in healthy adult participants.**

**
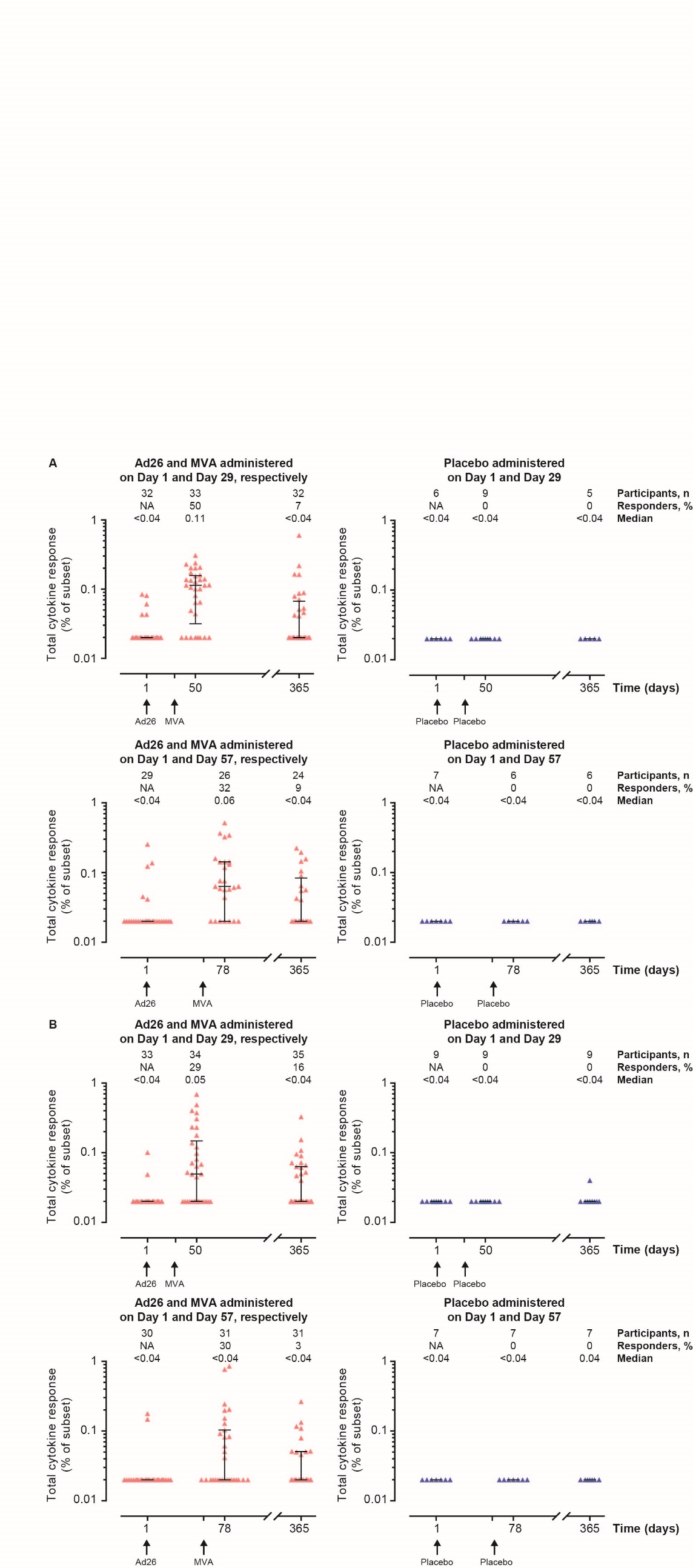
**

The vertical lines represent the IQR (Q1–Q3), where the lower and upper horizontal lines depict the lower quartile (Q1) and upper quartile (Q3), respectively. The middle horizontal line depicts the median (Q2).

Vaccines: Ad26 = Ad26.ZEBOV at a dose of 5x10^10^ vp; MVA = MVA-BN-Filo at a dose of 1x10^8^ Inf.U.

EBOV GP, Ebola virus glycoprotein; n, number of participants with data at that timepoint; NA, not applicable.
